# SITVITBovis—a publicly available database and mapping tool to get an improved overview of animal and human cases caused by *Mycobacterium bovis*

**DOI:** 10.1093/database/baab081

**Published:** 2022-01-13

**Authors:** David Couvin, Iñaki Cervera-Marzal, Audrey David, Yann Reynaud, Nalin Rastogi

**Affiliations:** WHO Supranational TB Reference Laboratory–TB and Mycobacteria Unit, Institut Pasteur de Guadeloupe, Abymes 97183, Guadeloupe, France; WHO Supranational TB Reference Laboratory–TB and Mycobacteria Unit, Institut Pasteur de Guadeloupe, Abymes 97183, Guadeloupe, France; WHO Supranational TB Reference Laboratory–TB and Mycobacteria Unit, Institut Pasteur de Guadeloupe, Abymes 97183, Guadeloupe, France; WHO Supranational TB Reference Laboratory–TB and Mycobacteria Unit, Institut Pasteur de Guadeloupe, Abymes 97183, Guadeloupe, France; WHO Supranational TB Reference Laboratory–TB and Mycobacteria Unit, Institut Pasteur de Guadeloupe, Abymes 97183, Guadeloupe, France

## Abstract

Limited data are available for bovine tuberculosis and the infections it can cause in humans and other mammals. We therefore constructed a publicly accessible SITVITBovis database that incorporates genotyping and epidemiological data on *Mycobacterium bovis*. It also includes limited data on *Mycobacterium caprae* (previously synonymous with the name *M. bovis* subsp. Caprae) that can infect both animals and humans. SITVITBovis incorporates data on 25,741 isolates corresponding to 60 countries of origin (75 countries of isolation). It reports a total of 1000 spoligotype patterns: 537 spoligotype international types (SITs, containing 25 278 clinical isolates) and 463 orphan patterns, allowing a wide overview of the geographic distribution of various phylogenetical sublineages (*BOV*_1, *BOV*_2, *BOV*_3 and *BOV*_4-*CAPRAE*). The SIT identifiers of the SITVITBovis were compared to the SB numbers of the Mbovis.org database to facilitate crosscheck among databases. Note that SITVITBovis also contains limited information on mycobacterial interspersed repetitive units-variable number of tandem repeats when available. Significant differences were observed when comparing age/gender of human isolates as well as various hosts. The database includes information on the regions where a strain was isolated as well as hosts involved, making it possible to see geographic trends. SITVITBovis is publicly accessible at: http://www.pasteur-guadeloupe.fr:8081/SITVIT_Bovis. Finally, a future second version is currently in progress to allow query of associated whole-genome sequencing data.

Database URLhttp://www.pasteur-guadeloupe.fr:8081/SITVIT_Bovis

## Introduction

Bovine tuberculosis (bTB) is an infectious disease caused by *Mycobacterium bovis*, a member of the *Mycobacterium tuberculosis* complex (MTBC). *M. bovis* alongside with *M. caprae* infect a wide range of mammalian hosts, including humans. An estimated 140 000 new cases of zoonotic tuberculosis (TB) and 11 400 deaths occurred globally in 2019 due to *M. bovis* (1). Knowing the difficulty to easily distinguish *M. bovis* from *M. tuberculosis*, these numbers possibly represent an underestimation of *M. bovis* cases worldwide.

According to the World Organisation for Animal Health (http://www.oie.int/en/), isolates of *M. bovis* or *M. caprae* were obtained from various animal species such as buffalos, bison, sheep, goats, horses, camels, pigs, wild boars, deer, antelopes, dogs, cats, foxes, minks, badgers, ferrets, rats, primates, llamas, kudu, eland, tapirs, elk, elephants, sitatungas, oryx, addax, rhinoceros, opossums, squirrels, otters, seals, hares, moles, raccoons, coyotes and several predatory felines including lions, tigers, leopards and lynx. Domestication of animals has long been a usual practice for mankind, and this practice seems to have caused irreversible effects in the evolution of bTB (2). Note that some animals could be infected by their own MTBC species (e.g. *Mycobacterium microti, Mycobacterium pinnipedii, Mycobacterium mungi, Dassie bacillus, Mycobacterium oryx* and *Mycobacterium leprae*). Details are provided in Supplementary File 1/Supplementary Table S1. Genotyping methods such as spoligotyping (3) and mycobacterial interspersed repetitive units-variable number of tandem repeats (MIRU-VNTRs) typing (4) have proven to be efficacious to distinguish the strains belonging to MTBC (5–7). Despite the limitations (such as homoplasy) of these polymerase chain reaction-based methods (8), these techniques are well used worldwide for MTBC family identification. Application of fingerprinting tools facilitates analysis of the molecular epidemiology of *M. bovis* in animal-to-human, human-to-human and even animal-to-animal transmission (9). In addition, studies based on whole-genome sequencing (WGS) analyses provide additional details which tend to become the norm in genomic epidemiology and evolutionary studies (10–12).

Development of worldwide or local databases dedicated to animal and human TB improves our global understanding on evolution and propagation of the disease. Mbovis.org database (www.Mbovis.org) (13) provides authoritative names for spoligotypes (SB numbers) of all members of MTBC isolates of animal origin. MycoDB.es (https://www.visavet.es/mycodb/) is another database focusing on zoonotic TB hosted in Spain (14).

The purpose of this study is to describe the SITVITBovis database containing available genotyping information on 25 741 *M. bovis* or *M. caprae* isolates. In addition to this information, preliminary data extracted from 188 raw sequence reads allowed to make some links with WGS and classical genotyping data. By publicly releasing this multimarker SITVITBovis database which incorporates user-friendly online tools and interfaces, we hope to serve the global research community with a concerted and coordinated response to monitor and assess global bTB spread.

## Materials and methods

### Ethics statement and collection of data

All the data (human and other mammalian isolates) were obtained from collaborating laboratories as described in SITVIT2 database (15). Data were duly de-identified prior to database entry. SITVITBovis is an excerpt from SITVIT2 database focusing on bTB isolates. It contains additional information on host (e.g. cattle, buffalo, deer, human, etc.) and $SB\_numbers$ collected from Mbovis.org database (13). Unlike Mbovis.org database that only provides spoligotypes, SITVITBovis provides available epidemiological data, such as information on host, WGS and MIRU-VNTRs. Many isolates were obtained from MycoDB.es study, accounting for more than 17 000 isolates at the time of the present study (16). Other isolates were obtained from other published studies (14, 17–20). SITVITBovis database aims to grow in the future and further updates will be applied, notably with the release of the upcoming SITVITEXTEND database. A user guide is provided online to facilitate navigation through the internet.

### Genotyping markers and WGS

The genotyping data included in SITVITBovis were similar to those previously described in SITVIT2 database (15). The methods used were spoligotyping (3) and MIRU-VNTRs typing, comprising 5-locus exact tandem repeats (ETR-A to E) (21) and 12- and 15-loci MIRU formats (4). The order of MIRU loci is as follows: 12-loci MIRU patterns—MIRU 2, 4, 10, 16, 20, 23, 24, 26, 27, 31, 39 and 40; 15-loci MIRU patterns—MIRU 4, 10, 16, 26, 31 and 40; ETR-A; ETR-C; QUB-11b; QUB-26; QUB-4156; Mtub04; Mtub21; Mtub30 and Mtub39. Spoligotype international type (SIT) indicates spoligotyping patterns found at least two times in database; VNTR international type (VIT) indicates 5-locus ETR patterns found at least two times in database; and 12- or 15-MIRU international type (12-MIT or 15-MIT) indicates 12- or 15-MIRU-VNTR patterns found at least two times in our database. Mbovis.org website was used to extract the SB numbers matching with some SIT numbers from SITVITBovis database (Supplementary Table S2). bTB sublineages (BOV, $BOV\_1$, $BOV\_2$, $BOV\_3$ and $BOV\_4-CAPRAE$) have been previously described in SITVIT classification using revised spoligotyping rules (7, 15). Note that BOV sublineage corresponds to previously labeled $BOV\_Like$ sublineage in SITVITWEB. To make a link with WGS data, we searched for genomes identified as ‘Mycobacterium bovis not BCG’ deposited in Sequence Read Archive (SRA) of the National Center for Biotechnology Information (NCBI) as suggested by a recent study (11). Then, an in-house pipeline algorithm (written in Perl language) was used to link WGS data extracted from the European Nucleotide Archive with classical genotyping information and sequencing-based tools. A total of 188 sequence reads were available with easily findable country information. The following tools were used for the analyses: SpoTyping, MIRUReader, SPAdes, SpolLineages, TBProfiler and Fast-lineage-caller (22–27). A preliminary strain list with read accessions is available on the ‘query’ web page. Formal presentation and query of WGS isolates will be available in an upcoming dedicated database which will contain more isolates.

### Computing approach for database construction

SITVITBovis is a publicly accessible website enabling the viewing of global spread of *M. bovis* isolates. It has been designed to work optimally with Google Chrome and Firefox. The web interface has been implemented using Java technology (Java Server Pages, Asynchronous JavaScript and Ajax), Google Code API and XML, under the integrated development environment Eclipse (https://www.eclipse.org/). Data were integrated within a MySQL database. Public access to the database is strictly on a read-only basis; therefore, no direct update of the database is allowed from this website. The web application is hosted and deployed on an Apache Tomcat Server (version 6).

### Phylogenetic, statistical and bioinformatics analyses

Existing bioinformatics tools have been used to realize the phylogenetic analysis. Minimum spanning trees (MSTs) based on spoligotypes or MIRU-VNTRs were drawn using BioNumerics software version 6.6 (Applied Maths, Sint-Martens-Latem, Belgium) or MLVA Compare version 1.03 software (GenoScreen; Lille, France). MSTs are undirected connected graphs which link all nodes (representing the isolates) together with the fewest possible linkages between nearest neighbors. Spoligoforests based on spoligotypes were drawn using the SpolTools software (28, 29); they allowed to describe and visualize the potential parent-to-descendant relationships among spoligotypes. In some cases, Spoligoforests have been colored and reshaped using GraphViz software (http://www.graphviz.org/). Contrary to the MST, the spoligoforest trees are directed graphs which only evolve by loss of spacers. In these trees, nodes are not necessarily all connected. In case of too many changes between two strains, there are no edges linking them. STATA software version 12 was used for descriptive and univariate analyses. Pearson’s chi-square test and Fisher’s exact test were used for comparison of different parameters and *P* values of <0.05 were considered as statistically significant. V-DICE tool was used to compare discriminatory powers of typing methods, providing Hunter–Gaston Diversity Index (HGDI) or Simpson’s diversity index (30).

## Results

### Structure and main functionalities of SITVITBovis

SITVITBovis was built based on the same architecture as SITVIT2 database. The users can browse through the web pages according to their needs. The following pages are accessible via the tabs ‘Database Description’, ‘Search’, ‘Analysis’, ‘Online Tools’, ‘Statistical Analysis’, ‘WGS data’ and ‘Links and Others’: (i) The ‘Database Description’ page provides general information about genotyping molecular markers contained in the database as well as global distribution of *M. bovis* strains and mammalian hosts represented in the database. (ii) The ‘Search’ page allows users to query SITVITBovis database according to several criteria (such as spoligotype, 12-loci MIRU, SIT, SB number, 12-MIT, lineage, isolation country, drug resistance, host, etc.). Regular expressions can be used. (iii) The ‘Analysis’ page allows users to analyze their own data based on the example file provided on the web page. (iv) The ‘Online Tools’ page allows users to get information on the distribution of isolates in function of several characteristics such as genotyping markers, lineages, hosts, country or city of isolation, etc. (v) The ‘Statistical Analysis’ provides information on the evolution of strains over time and space in function of hosts. (vi) The ‘WGS data’ page contains a preliminary strain list linking read accessions with information on classical genotyping data and drug resistance. (vii) The ‘Links and Others’ page provides supplemental information on interesting links and lab.

### Worldwide diversity of bTB genotypes

**Figure 1.**
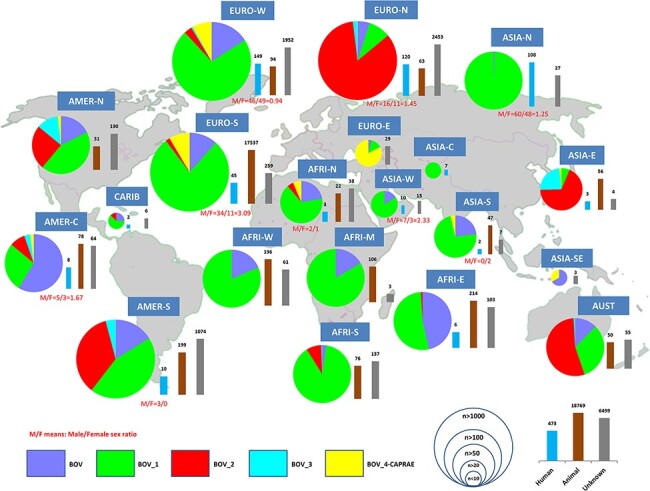
Worldwide distribution of bTB isolates and their human or animal hosts by United Nations (UN) sub-region.

The SITVITBovis database provides a broad overview of the geographic distribution of various phylogenetical sublineages belonging to *M. bovis* ($BOV\_1$, $BOV\_2$, $BOV\_3$ and $BOV\_4-CAPRAE$). This web application also helps distinguish between strains isolated from humans and other mammals in order to better understand transmission pathways and other factors that could potentially be responsible for the global spread of bTB. In our study, among the 25 741 isolates, $BOV\_1$ sublineage was globally the most predominant, representing 67.7% ($n=17\,427$) of bTB clinical isolates, followed by $BOV\_2$ ($n=3258$ or 12.7%), BOV ($n=3157$ or 12.3%), $BOV\_4-CAPRAE$ ($n=1735$ or 6.7%) and $BOV\_3$ ($n=164$ or 0.6% of isolates). (i) $BOV\_1$ was notably predominantly found (number of isolates $\geq 25$) in Russia, Western and Southern Europe, the whole African continent (North, Middle, West, Austral and East), the Middle-East (Western Asia), Southern Asia and North and South America, with proportions between 43% and 99% (Figure 1). (ii) $BOV\_2$ sublineage was predominantly found in Northern Europe (84% of clinical isolates), Eastern Asia (68%), Australasia (54%), South America (35%), North America (25%), Central America (9%) and Austral Africa (8%) (Figure 1). (iii) $BOV _3$ sublineage was scarcely found in our study, being limited to East Asia (24%), North America (12%) and South America (4%). (iv) $BOV\_4-CAPRAE$ lineage was predominantly found in Eastern Europe (representing 83% of isolates). This lineage represented 8% of clinical isolates in Western Europe and South America and 6% of the isolates in North Africa. (v) BOV sublineage was predominantly found in Central America (59% of isolates), followed by East Africa (46% of strains), and with proportions from 12% to 23% in North, West and Central Africa; North and South America; Western and Southern Europe; and Western and Southern Asia (Figure 1).

The database provided information on strains belonging to human hosts ($n=473$ isolates) and other mammalian hosts (mainly cattle; $n= 18\,769$ isolates). Most of the bTB isolates from animals were found in Southern Europe (predominantly in Spain), whereas available isolates from humans were mainly found in Western Europe ($n=149$), Northern Europe ($n=120$) and followed by Northern Asia or Russia ($n=108$) (Figure 1). Among human isolates, gender of patients was known for 301 isolates, and the global male/female sex ratio was $173/128 = 1.35$. In countries where at least 10 human isolates were recorded, the highest sex ratio was observed in Southern Europe (male/female sex ratio=3.09), as opposed to the lowest ratio in Western Europe (male/female sex ratio=0.94). Regarding the distribution of lineages between human and animal hosts (Supplementary File 1/Supplementary Figure S1), $BOV\_1$ sublineage was globally more common among human hosts ($n=388/473$; 82.03%), followed by BOV sublineage ($n=72/473$; 15.22%). Considering the diverse sub-regions, BOV sublineage that predominated among animal isolates in Central America (around 88% of isolates) was also largely present among patients from Northern Europe (around 36% of isolates). Lastly, the $BOV\_3$ sublineage was merely visible in patients from Eastern Asia ($n=2$) and South America ($n=1$). Table 1 shows the distribution of predominant spoligotypes in SITVITBovis.

**Table 1.** Distribution of predominant spoligotyping patterns representing at least 1% of isolates in SITVITBovis

**SIT/SB number** **(Lineage)**	**Spoligotype Description (Octal code)**	**Number of strains (%)**	**Percentage distribution by UN sub-region**	**Percentage distribution by country**
481/SB0121 (BOV_1)	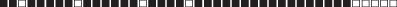 (676 773 677 777 600)	5338 (20.74)	EURO-S 89.75, EURO-W 5.38, AMER-S 2.59, AFRI-S 1.78, AMER-N 0.15, AMER-C 0.15, EURO-N 0.09, AFRI-N 0.08	ESP 89.62, FXX 3.09, BRA 1.86, ZAF 1.78, NLD 1.12, DEU 0.77, ARG 0.71, BEL 0.39, USA 0.15, MEX 0.15, ITA 0.09, SWE 0.06, TUN 0.04, PRT 0.04, GBR 0.04, MAR 0.02, DZA 0.02
683/SB0140 (BOV_2)	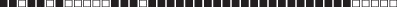 (664 073 777 777 600)	2215 (8.60)	EURO-N 65.69, AMER-S 17.83, EURO-S 8.4, EURO-W 2.48, ASIA-E 1.94, AUST 1.9, AFRI-S 0.81, AMER-N 0.41, AMER-C 0.36, AFRI-E 0.14, CARI 0.05	IRL 44.88, GBR 20.81, ARG 14.94, ESP 7.09, KOR 1.94, BRA 1.94, NLD 1.67, PRT 1.08, NZL 1.08, AUS 0.81, ZAF 0.77, CHL 0.72, FXX 0.73, USA 0.41, MEX 0.36, ITA 0.23, MOZ 0.14, PRY 0.09, BEL 0.09, SWZ 0.05, MTQ 0.05
665/SB0134 (BOV_1)	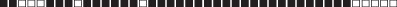 (616 773 777 777 600)	2057 (7.99)	EURO-S 92.76, EURO-W 5.35, AMER-S 0.49, AFRI-E 0.39, AFRI-W 0.29, AFRI-S 0.19, EURO-N 0.15, AFRI-N 0.15	ESP 92.12, FXX 4.03, BEL 1.02, ITA 0.63, BRA 0.44, MLI 0.29, ETH 0.29, ZAF 0.19, NLD 0.15, GBR 0.15, DEU 0.15, TUN 0.1, REU 0.1, DZA 0.05, ARG 0.05
482/SB0120 (BOV_1)	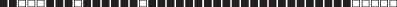 (676 773 777 777 600)	1661 (6.45)	EURO-S 39.49, EURO-W 35.88, ASIA-N 8.07, EURO-N 5.54, AMER-S 3.61, AMER-N 1.69, ASIA-S 1.26, ASIA-W 1.2, AFRI-N 1.2, AFRI-E 0.36, CARI 0.3, AFRI-S 0.3, EURO-E 0.24, AMER-C 0.18, AUST 0.12, ASIA-E 0.12	ESP 34.26, FXX 30.36, RUS 8.07, ITA 5.06, SWE 3.31, NLD 3.19, DEU 3.13, BRA 2.05, DNK 1.75, USA 1.69, ARG 1.33, IRN 1.26, SAU 0.72, AUT 0.72, TUN 0.6, DZA 0.6, BEL 0.48, GBR 0.42, OMN 0.36, ZAF 0.24, ZMB 0.18, PRT 0.18, MEX 0.18, GLP 0.18, PSE 0.12, NZL 0.12, JAM 0.12, GUF 0.12, ETH 0.12, CZE 0.12, BGR 0.12, VEN 0.06, TWN 0.06, SUR 0.06, NOR 0.06, NAM 0.06, MOZ 0.06, KOR 0.06
696/SB0339 (BOV_1)	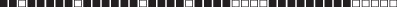 (676 773 674 177 600)	1371 (5.33)	EURO-S 98.47, EURO-W 1.53	ESP 98.47, FXX 1.17, NLD 0.15, DEU 0.07, BEL 0.07, AUT 0.07
684/SB0265 (BOV_1)	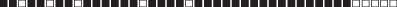 (666 773 677 777 600)	960 (3.73)	EURO-S 96.46, EURO-W 1.88, AMER-N 0.73, AFRI-S 0.63, ASIA-E 0.21, AFRI-N 0.1	ESP 96.25, FXX 1.04, USA 0.73, ZAF 0.63, BEL 0.42, NLD 0.31, TWN 0.21, TUN 0.1, PRT 0.1, ITA 0.1, DEU 0.1
698/SB0295 (BOV_1)	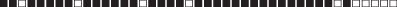 (676 773 677 777 200)	949 (3.69)	EURO-S 86.62, AMER-S 11.7, EURO-W 1.05, AMER-C 0.53, AMER-N 0.11	ESP 86.09, BRA 10.22, ARG 1.48, PRT 0.53, FXX 0.53, MEX 0.42, NLD 0.32, BEL 0.21, USA 0.11, CRI 0.11
691/SB0130 (BOV_1)	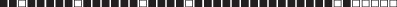 (676 573 777 777 600)	788 (3.06)	EURO-S 67.01, AMER-S 14.09, AFRI-S 5.33, EURO-N 5.2, AUST 3.93, EURO-W 1.78, AMER-N 1.65, AMER-C 0.51, AFRI-N 0.38, ASIA-W 0.13	ESP 66.5, ARG 10.79, ZAF 5.33, NZL 3.93, BRA 2.92, IRL 2.67, USA 1.65, SWE 1.27, GBR 1.27, FXX 1.65, MEX 0.51, TUN 0.38, PRY 0.38, PRT 0.38, TUR 0.13, NLD 0.13, ITA 0.13
645/SB0157 (BOV_4-CAPRAE)	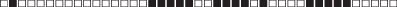 (200 003 717 207 600)	744 (2.89)	EURO-S 99.87, EURO-W 0.13	ESP 99.87, NLD 0.13
679/SB0142 (BOV_2)	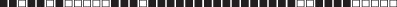 (664 073 777 763 600)	431 (1.67)	EURO-N 99.07, EURO-W 0.46, AMER-S 0.46	IRL 73.32, GBR 25.75, NLD 0.46, ARG 0.46
980/SB0152 (BOV)	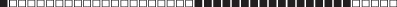 (400 000 077 777 600)	356 (1.38)	EURO-S 95.23, EURO-W 4.78	ESP 95.23, FXX 4.49, NLD 0.28
695/SB0119 (BOV)	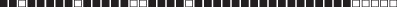 (676 763 677 777 600)	294 (1.14)	EURO-S 96.6, EURO-W 2.04, AMER-C 0.68, AMER-S 0.34, AMER-N 0.34	ESP 96.6, NLD 1.02, MEX 0.68, DEU 0.68, USA 0.34, BRA 0.34, BEL 0.34
646/SB0416 (BOV_4-CAPRAE)	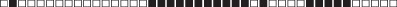 (200 003 777 207 600)	290 (1.13)	EURO-S 97.93, EURO-W 2.07	ESP 94.14, ITA 3.79, DEU 1.38, FXX 0.69

The black square denotes the presence of a spacer while the white square denotes the absence of a spacer.

Note that the country or UN sub-region (http://unstats.un.org/unsd/methods/m49/m49regin.htm) of isolation of some strains was not known (this explains why the global distribution of isolates is not always exhaustive or equal to 100%). The 3-letter country codes are according to *http://en.wikipedia.org/wiki/ISO_3166-1_alpha-3*. FXX country code designates Metropolitan France.

Globally, the four genotyping methods (spoligotyping, 5-locus ETRs and 12- and 15-loci MIRU-VNTRs) showed good discriminatory power (HGDI in range of 0.930–0.979; Table 2). Regarding the individual VNTR loci, we noted that QUB-11b (VNTR2163), ETR-A (VNTR2165), MIRU16 (VNTR1644) and MIRU04 (VNTR0580) displayed reasonable discriminatory power with HGDI>0.6 (Table 2).

**Table 2.** Diversity of spoligotypes and MIRU-VNTR patterns and loci

**Spoligotyping and** **MIRU-VNTRs** **scheme or loci**	**Hunter–Gaston Diversity (95% confidence interval)**	**Simpson’s diversity (95% confidence interval)**
12-loci MIRU-VNTRs	0.973 (0. 967–0.978)	0.970 (0. 964–0.975)
MIRU16 (VNTR)*	0.623 (0. 589–0.657)	0.621 (0. 587–0.655)
MIRU04 (VNTR)*	0.618 (0. 575–0.662)	0.616 (0. 573–0.660)
MIRU31 (VNTR)*	0.537 (0. 487–0.587)	0.535 (0. 485–0.585)
MIRU10 (VNTR)*	0.534 (0. 481–0.587)	0.532 (0. 479–0.585)
MIRU26 (VNTR)*	0.437 (0. 376–0.498)	0.436 (0. 375–0.496)
MIRU23 (VNTR)	0.265 (0. 200–0.329)	0.264 (0. 199–0.328)
MIRU27 (VNTRc)	0.259 (0. 195–0.323)	0.258 (0. 194–0.322)
MIRU24 (VNTR)	0.099 (0. 052–0.146)	0.099 (0. 052–0.146)
MIRU40 (VNTR)*	0.072 (0. 034–0.109)	0.071 (0. 034–0.109)
MIRU20 (VNTR)	0.073 (0. 032–0.114)	0.073 (0. 032–0.114)
MIRU02 (VNTR)	0.034 (0. 005–0.063)	0.034 (0. 005–0.063)
MIRU39 (VNTR)	0.000 (0. 000–0.025)	0.000 (0. 000–0.025)
15-loci MIRU-VNTRs	0.979 (0. 962–0.996)	0.964 (0. 947–0.981)
QUB-11b (VNTR)	0.668 (0. 613–0.723)	0.658 (0. 603–0.713)
ETR-A (VNTR)*	0.653 (0. 611–0.695)	0.651 (0. 610–0.693)
QUB-4156 (VNTR)	0.544 (0. 450–0.639)	0.536 (0. 441–0.630)
ETR-C (VNTR)*	0.481 (0. 430–0.532)	0.480 (0. 429–0.531)
QUB-26 (VNTR)	0.476 (0. 374–0.578)	0.469 (0. 367–0.571)
Mtub21 (VNTR)	0.455 (0. 339–0.571)	0.448 (0. 332–0.564)
Mtub04 (VNTR)	0.441 (0. 336–0.546)	0.434 (0. 329–0.539)
Mtub30 (VNTR)	0.433 (0. 334–0.532)	0.426 (0. 327–0.525)
Mtub39 (VNTR)	0.092 (0. 000–0.188)	0.090 (0. 000–0.186)
5-locus ETRs (VNTR)	0.930 (0. 914–0.947)	0.928 (0. 911–0.944)
ETR-B (VNTR)	0.559 (0. 503–0.615)	0.558 (0. 502–0.614)
ETR-D	0.237 (0. 180–0.294)	0.236 (0. 179–0.293)
ETR-E	0.111 (0. 067–0.154)	0.111 (0. 067–0.154)
Spoligotypes (43 spacers)	0.930 (0. 928–0.932)	0.930 (0. 928–0.932)

* Common loci between 5-locus ETRs and 12- and 15-loci MIRUs.

Spoligoforest trees (Supplementary File 2) were drawn to highlight diversity of main spoligotypes (represented by a SB number or a SIT) in Africa ($n=965$), The Americas ($n=1662$), Asia ($n=289$), Australasia ($n=105$), and Europe ($n=22701$). In these trees, we can visualize the main spoligotypes in each continent. One may notice that, according to our database, SB0121/SIT481 was more predominant in Europe ($n=5083$ isolates), followed by SB0134/SIT665 ($n=2021$), and SB0140/SIT683 ($n=1696$); while SB0140/SIT683 ($n=42$) predominated in Australasia, followed by SB0130/SIT691 ($n=31$); SB0120/SIT482 was more prevalent in Asia ($n=179$), followed by SB0140/SIT683 ($n=85$) and SB0130/SIT691 ($n=32$); SB0140/SIT683 was more predominant in the Americas ($n=413$), followed by SB0121/SIT481 ($n=154$), and SB0130/SIT691 ($n=128$); finally, SB0944/SIT1037 was more predominant in Africa ($n=136$), followed by SB0121/SIT481 ($n=99$) and SB1176/SIT3453 ($n=59$). Besides, a supplementary file was added in order to allow comparisons between different regions (Supplementary File 3).

### Proposal of an international consensus schema based on MIRU-VNTR loci

Several studies have reported affordable and suitable sets of VNTR loci to discriminate bTB isolates (31–33). However, most of these studies have been based on specific countries. In SITVITBovis database, we have gathered data from various countries, providing a wider view of bTB diversity. According to pooled data in our database, an optimal consensus set could be based on 13 MIRU-VNTR loci showing reasonably high discriminatory power (HGDI>0.4), i.e. QUB-11b (VNTR2163), ETR-A (VNTR2165), MIRU16 (VNTR1644), MIRU04 (VNTR0580), ETR-B (VNTR2461), QUB-4156 (VNTR4156), MIRU31 (VNTR3192), MIRU10 (VNTR0960), ETR-C (VNTR0577), QUB-26 (VNTR4052), Mtub21 (VNTR1955), Mtub04 (VNTR0424) and Mtub30 (VNTR2401) (Table 2).

In addition to the aforementioned VNTRs, we also recommend to include QUB3232 (VNTR3232) as suggested earlier by other studies (31, 32, 34), to further increase the discriminatory power in bTB diversity analyses.

### Distribution of patient age groups

Comparison with SITVIT2 database showed that the distribution of patient age groups of *M. bovis* isolates (belonging to $BOV\_1$ sublineage) was different from age group distributions in patients infected with other MTBC members (*P*-value < 0.0001). When comparing the distribution of age groups 0–20 years, 21–40 years, 41–60 years and >60 years among patient isolates belonging to H3, LAM9, T1, Beijing, CAS1-Delhi and EAI5 sublineages, we noticed that patient isolates belonging to $BOV\_1$ sublineage were more common among younger (age group 0–20 years) as well as older patients (>60 years) (Table 3).

**Table 3.** Comparison of distributions of age groups among isolates of patients infected by BOV_1 sublineage as compared to other predominant sublineages found in SITVIT2 database

**Lineage**	**Number of strains**	**%0–20 yrs**	**%21–40 yrs**	**%41–60 yrs**	**%>60 yrs**
Beijing	2640	9.51	39.92	22.20	28.37
BOV_1	162	22.22	25.31	15.43	37.04
CAS1-Delhi	1085	18.53	50.05	22.30	9.12
EAI5	393	12.21	51.15	26.46	10.18
H3	1369	11.91	37.84	22.50	27.76
LAM9	1333	11.78	46.59	25.58	16.05
T1	2641	11.09	39.57	22.95	26.39

Considering only the bTB strains isolated in Europe (north, south and west, with most of the available data), we noted a significant disparity in the distribution of age groups (*P*-value < 0.0001). For all age groups (0–20 years, 21–40 years, 41–60 years and >60 years), we observed that the proportion of patients in Western Europe (EURO-W) was similar in each age group, representing around 20% (from 18 to 24%) of cases, except for the age group >60 years which represented about 35% of patients (Supplementary File 1/Supplementary Figure S2A). On the contrary, a majority of patients in Southern Europe (EURO-S) belonged to the age group 21–40 years (51% of patients), as opposed to the age group >60 years (81% of cases) in Northern Europe (EURO-N). These observations suggest that bovine tuberculosis is a major problem primarily affecting the working population in Southern Europe, while it rather concerns reactivation cases among senior patients in Northern Europe.

## Discussion

Results obtained from this study suggested an important heterogeneity in worldwide distribution of bTB isolates. Both significant cleavages and similarities were observed in the geographical distribution of various bTB sublineages defined in our database as well as for the various bTB genotypes.

Diversity, presence and/or absence of certain spoligotyping spacers have been revealed as being particularly useful for discriminating specific bTB isolates circulating worldwide (16, 13). Our web-based tool allows to have a global overview of bTB genotypes (including spoligotypes, E-locus ETRs and 12- and 15-loci MIRU-VNTRs). By using SITVITBovis tool, users can remarkably visualize the geographical position (at sub-region, country or city level) of specific genotypes in addition to available epidemiological data as well as drug resistance information; the latter being an important health issue in various regions of the world (35).

MIRU-VNTR method obviously increases the discriminatory power of spoligotyping; nonetheless, spoligotyping alone is still a reliable method to differentiate *M. bovis* isolates (Table 2). Several studies have focused on the diversity of MIRU-VNTRs and/or spoligotypes involved in bTB (31, 32). However, the majority of these studies generally focused on limited geographical areas.

In our analysis, spoligotyping was globally considered as a reasonable typing method to discriminate bTB isolates (with a global HGDI of 0.930). However, according to recent research, its discriminatory power could vary in function of countries (33). Despite an international consensus scheme of using MIRU-VNTR loci concomitantly with spoligotyping for improved characterization of MTBC (4–6, 15), only selected groups working on bTB isolates have used such an approach (31–34). Henceforth, the scarcity of MIRU-VNTR data in SITVITBovis should be improved by adding new data in future updates. Nonetheless, with data on 25 741 isolates (75 countries of isolation corresponding to 60 countries of origin), SITVITBovis still incorporates valuable data from other resources. Unfortunately, information on host is not always provided for all collected bTB isolates in published literature, which hampers efforts to precisely calculate the real incidence of bTB among human vs. other animal hosts. Due to the lack of available information, we did not address the issues of clonal complexes of *M. bovis* marked by specific regions of deletions (RDs) or single-nucleotide polymorphisms (SNPs) in the present study. Nevertheless, information on RDs may be obtained by querying the database and exporting results into an Excel file. Last but not least, WGS and SNPs are being frequently used today to decipher bTB transmission and identify lineages involved (36, 37). Studies using WGS already provide significant insights in bTB diversity, distribution and evolution (10–12) and may help identify hosts that could serve as reservoir of bTB infection and spread. As an illustration, a preliminary table linking WGS and classical genotyping data for some bTB isolates is available (Supplementary Table S3).

As the genomic information becomes more easily accessible in conjunction with geographical origin and hosts, we plan to automatically extract from sequencing data a number of variables such as specific phylogenetic markers, drug resistance, genomic determinants of virulence/pathogenicity or other relevant information. This novel generation of database(s) will be able to link the newly generated information with the existing data on classical genotyping information vs. hosts and geographical mapping. The development of all these tools in future bTB genotyping databases would possibly allow anticipating bTB outbreaks among humans and animal hosts alike.

## Conclusion and perspectives

In summary, our study provides a global overview of bTB distribution, highlighting potential relationships between bTB genotypes and affected hosts as well as other epidemiological features. SITVITBovis, with its correlation with other existing resources (such as Mbovis.org), provides a tentative picture of bTB circulation in the world and therefore represents a non-negligible resource for monitoring bTB and make correlations between epidemiological, macro-geographical and other data available.

Future developments aim to enrich the database with WGS data in conjunction with geographical origin and hosts. Development of automated extraction of knowledge related to specific phylogenetic markers, drug resistance, genomic determinants of virulence/pathogenicity or other relevant information will soon make it possible to link the newly generated information with the existing data generated using classical genotyping tools. The development of such a strategy in conjunction with information on hosts and subsequent geographical mapping will boost our efforts to generate a real comprehensive snapshot of global bTB diversity. Being helpful to better identify, treat and control bTB, such a tool will be highly beneficial for both medical and veterinarian specialists as well as public health authorities.

## Supplementary Material

baab081_Supp
