# Supplementary material for: SITVITBovis—a publicly available database and mapping tool to get an improved overview of animal and human cases caused by Mycobacterium bovis
Source: Database (Oxford). 2022 Jan 13;2022:baab081. doi: 10.1093/database/baab081 (PMC8962452; doi:10.1093/database/baab081)
Supplement: baab081_Supp [file baab081_supp.zip › sitvitbovis_database.pdf]

Database, 2021, 1–9
doi: DOI HERE
Advance Access Publication Date: Day Month Year
Paper

Subject Section

SITVITBovis - a publicly available database and mapping tool to get an improved overview of animal and human cases caused by Mycobacterium bovis

David Couvin,[1,*] Iñaki Cervera-Marzal,[1] Audrey David,[1] Yann Reynaud[1] and Nalin Rastogi[1,*]

[1]WHO Supranational TB Reference Laboratory – TB & Mycobacteria Unit, Institut Pasteur de Guadeloupe, Abymes, 97183, Guadeloupe, France

*Corresponding authors. dcouvin@pasteur-guadeloupe.fr ; nrastogi@pasteur-guadeloupe.fr

Associate Editor: Name

FOR PUBLISHER ONLY Received on Date Month Year; revised on Date Month Year; accepted on Date Month Year

Abstract

Limited data are available for bovine tuberculosis (bTB) and the infections it can cause in humans and other mammals. We therefore constructed a publicly accessible SITVITBovis database that incorporates genotyping and epidemiological data on *M. bovis*. It also includes limited data on *Mycobacterium caprae* (previously synonymous with the name *Mycobacterium bovis* subsp. Caprae) that can infect both animals and humans. SITVITBovis incorporates data on 25,741 isolates corresponding to 60 countries of origin (75 countries of isolation). It reports a total of 1000 spoligotype patterns: 537 spoligotype international types (SITs, containing 25,278 clinical isolates) and 463 orphan patterns, allowing a wide overview of the geographic distribution of various phylogenetical sub-lineages (BOV_1, BOV_2, BOV_3, $BOV\_4 - CAPRAE$). The SIT identifiers of the SITVITBovis were compared to the SB numbers of the Mbovis.org database to facilitate crosscheck among databases. Note that SITVITBovis also contains limited information on mycobacterial interspersed repetitive units-variable number of tandem repeats (MIRU-VNTR) when available. Significant differences were observed when comparing age/gender of human isolates, as well as various hosts. The database includes information on the regions where a strain was isolated as well as hosts involved, making it possible to see geographic trends. SITVITBovis is publicly accessible at: http://www.pasteur-guadeloupe.fr:8081/SITVIT_Bovis. Finally, a future 2nd version is currently in progress to allow query of associated WGS data.

Key words: *Mycobacterium bovis*, Database, Genotyping, Spoligotyping, MIRU-VNTR, Epidemiology, Host, Whole genome sequencing

Introduction

Bovine tuberculosis (bTB) is an infectious disease caused by *Mycobacterium bovis*, a member of the *Mycobacterium tuberculosis* complex (MTBC). *M. bovis* alongside with *M. caprae*, infect a wide range of mammalian hosts, including humans. An estimated 140 000 new cases of zoonotic TB and 11 400

© The Author 2021. Published by Oxford University Press. All rights reserved. For permissions, please e-mail: journals.permissions@oup.com

deaths occurred globally in 2019 due to *M. bovis* [1]. Knowing the difficulty to easily distinguish *M. bovis* from *M. tuberculosis*, these numbers possibly represent an underestimation of *M. bovis* cases worldwide.

According to the World Organisation for Animal Health (http://www.oie.int/en/), isolates of *M. bovis* or *M. caprae* were obtained from various animal species such as: buffalos, bison, sheep, goats, horses, camels, pigs, wild boars, deer, antelopes, dogs, cats, foxes, minks, badgers, ferrets, rats, primates, llamas, kudu, eland, tapirs, elk, elephants, sitatungas, oryx, addax, rhinoceros, opossums, squirrels, otters, seals, hares, moles, raccoons, coyotes and several predatory felines including lions, tigers, leopards and lynx. Domestication of animals has long been a usual practice for mankind, and this practice seems to have caused irreversible effects in the evolution of bTB [2]. Note that some animals could be infected by their own MTBC species (e.g. *M. microti, M. pinnipedii, M. mungi, Dassie bacillus, M. oryx and M. leprae*). Details were provided in Supplementary File 1/Supplementary Table S1. Genotyping methods such as spoligotypting [3] and mycobacterial interspersed repetitive units-variable number of tandem repeats (MIRU-VNTRs) typing [4] have proven to be efficacious to distinguish the strains belonging to MTBC [5, 6, 7]. Despite the limitations (such as homoplasy) of these PCR-based methods [8], these techniques are well used worldwide for MTBC family identification. Application of fingerprinting tools facilitates analysis of the molecular epidemiology of *M. bovis* in animal-to-human, human-to-human, and even animal-to-animal transmission [9]. In addition, studies based on WGS analyses provide additional details which tend to become the norm in genomic epidemiology and evolutionary studies [10, 11, 12].

Development of worldwide or local databases dedicated to animal and human tuberculosis improves our global understanding on evolution and propagation of the disease. Mbovis.org database (www.Mbovis.org) [13] provides authoritative names for spoligotypes (SB numbers) of all members of MTBC isolates of animal origin. MycoDB.es (https://www.visavet.es/mycodb/) is another database focusing on Zoonotic tuberculosis hosted in Spain [14].

The purpose of this study is to describe the SITVITBovis database containing available genotyping information on 25,741 *M. bovis* or *M. caprae* isolates. In addition to this information, preliminary data extracted from 188 raw sequence reads allowed to make some links with whole genome sequencing (WGS) and classical genotyping data. By publicly releasing this multimarker SITVITBovis database which incorporates user-friendly online tools and interfaces, we hope to serve the global research community with a concerted and coordinated response to monitor and assess global bTB spread.

Materials and Methods

Ethics statement and collection of data

All the data (human and other mammalian isolates) were obtained from collaborating laboratories as described in SITVIT2 database [15]. Data were duly de-identified prior to database entry. SITVITBovis is an excerpt from SITVIT2 database focusing on bTB isolates. It contains additional information on host (e.g. cattle, buffalo, deer, human, etc.) and *SB_numbers* collected from Mbovis.org database [13]. Unlike Mbovis.org database that only provides spoligotypes, SITVITBovis provides available epidemiological data, such as information on host, WGS, and MIRU-VNTRs. Many isolates were obtained from MycoDB.es study accounting for more than 17,000 isolates at the time of the present study [16]. Other isolates were obtained from other published studies [14, 17, 18, 19, 20]. SITVITBovis database aims to grow in the future and further updates will be applied, notably with the release of the upcoming SITVITEXTEND database. A user guide is provided online to facilitate navigation through the internet.

Genotyping markers and whole genome sequencing (WGS)

The genotyping data included in SITVITBovis were similar to those previously described in SITVIT2 database [15]. The methods used were spoligotypting [3] and MIRU-VNTRs typing, comprising 5-locus exact tandem repeats (ETR-A to E) [21], and 12- and 15-loci MIRU formats [4]. The order of MIRU loci is as follows: 12-loci MIRU patterns: MIRU 2, 4, 10, 16, 20, 23, 24, 26, 27, 31, 39 and 40; 15-loci MIRU patterns : MIRU 4, 10, 16, 26, 31, and 40, ETR-A, ETR-C, QUB-11b, QUB-26, QUB-4156, Mtub04, Mtub21, Mtub30, and Mtub39. Spoligotype International Type (SIT) indicates spoligotyping patterns found at least two times in database; VNTR International Type (VIT) indicates 5-locus ETRs patterns found at least two

times in database; and 12- or 15-MIRU International Type (12-MIT or 15-MIT) indicates 12- or 15-MIRU-VNTRs patterns found at least two times in our database. Mbovis.org website was used to extract the SB numbers matching with some SITs numbers from SITVITBovis database (Supplementary Table S2). bTB sublineages (BOV, BOV_1, BOV_2, BOV_3, $BOV\_4-CAPRAE$) have been previously described in SITVIT classification using revised spoligotyping rules [7, 15]. Note that BOV sublineage corresponds to previously labelled BOV_Like sublineage in SITVITWEB. To make a link with WGS data, we searched for genomes identified as "Mycobacterium bovis not BCG" deposited in SRA, NCBI as suggested by a recent study [11]. Then, an in-house pipeline algorithm (written in Perl language) was used to link WGS data extracted from the European Nucleotide Archive (ENA) with classical genotyping information and sequencing based tools. A total of 188 sequence reads were available with easily findable country information. The following tools were used for the analyses: SpoTyping, MIRUReader, SPAdes, SpolLineages, TBProfiler, and Fast-lineage-caller [22, 23, 24, 25, 26, 27]. A preliminary strain list with read accessions is available on the "query" web page. Formal presentation and query of WGS isolates will be available in an upcoming dedicated database which will contain more isolates.

Computing approach for database construction

SITVITBovis is a publicly accessible website enabling the viewing of global spread of *M. bovis* isolates. It has been designed to work optimally with Google Chrome and Firefox. The web interface has been implemented using Java technology (Java Server Pages, Asynchronous JavaScript, Ajax), Google Code API, and XML, under the integrated development environment (IDE) Eclipse (https://www.eclipse.org/). Data were integrated within a MySQL database. Public access to the database is strictly on a read only basis, therefore, no direct update of the database is allowed from this website. The web application is hosted and deployed on an Apache Tomcat Server (version 6).

Phylogenetic, statistical and bioinformatics analyses

Existing bioinformatics tools have been used to realize the phylogenetic analysis. Minimum spanning trees (MSTs) based on spoligotypes or MIRU-VNTRs were drawn using BioNumerics software version 6.6 (Applied Maths, Sint-Martens-Latem, Belgium) or MLVA Compare version 1.03 software (GenoScreen; Lille, France). MSTs are undirected connected graphs which link all nodes (representing the isolates) together with the fewest possible linkages between nearest neighbours. Spoligoforests based on spoligotypes were drawn using the SpolTools software [28, 29], they allowed to describe and visualize the potential parent-to-descendant relationships among spoligotypes. In some cases, Spoligoforests have been coloured and reshaped using GraphViz software (http://www.graphviz.org/). Contrarily to the MST, the spoligoforest trees are directed graphs which only evolve by loss of spacers. In these trees, nodes are not necessarily all connected. In case of too many changes between two strains, there are no edges linking them. STATA software version 12 was used for descriptive and univariate analyses. Pearson's Chi-square test and Fisher's Exact Test were used for comparison of different parameters and P values of <0.05 were considered as statistically significant. V-DICE tool was used to compare discriminatory powers of typing methods, providing Hunter-Gaston Diversity Index (HGDI) or Simpson's diversity index [30].

Results

Structure and main functionalities of SITVITBovis

SITVITBovis was built based on the same architecture as SITVIT2 database. The users can browse through the web pages according to their needs. The following pages are accessible via the tabs "Database Description", "Search", "Analysis", "Online Tools", "Statistical Analysis", "WGS data", and "Links and Others": (i) The "Database Description" page provides general information about genotyping molecular markers contained in the database, as well as global distribution of *M. bovis* strains, and mammalian hosts represented in the database. (ii) The "Search" page allows users to query SITVITBovis database according to several criteria (such as Spoligotype, 12-loci MIRU, SIT, SB-number, 12-MIT, Lineage, Isolation Country, Drug resistance, Host, etc.). Regular expressions can be used. (iii) The "Analysis" page allows users to analyze their own data based on the example file provided on the web page. (iv) The "Online Tools" page allows users to get information on the distribution of isolates in function of several

characteristics such as genotyping markers, lineages, hosts, country or city of isolation, etc. (v) The "Statistical Analysis" provides information on the evolution of strains over time and space in function of hosts. (vi) The "WGS data" page contains a preliminary strain list linking read accessions with information on classical genotyping data and drug resistance. (vii) The "Links and Others" page provides supplemental information on interesting links and lab.

Worldwide diversity of bTB genotypes

The SITVITBovis database provides a broad overview of the geographic distribution of various phylogenetical sub-lineages belonging to *M. bovis* (BOV_1, BOV_2, BOV_3, $BOV\_4-CAPRAE$). This web application also helps distinguish between strains isolated from humans and other mammals in order to better understand transmission pathways, and other factors that could potentially be responsible for the global spread of bTB. In our study, among the 25,741 isolates, BOV_1 sub-lineage was globally the most predominant, representing 67.7% (n=17427) of bTB clinical isolates, followed by BOV_2 (n=3258 or 12.7%), BOV (n=3157 or 12.3%), $BOV\_4-CAPRAE$ (n=1735 or 6.7%), and BOV_3 (n=164 or 0.6% of isolates). (i) BOV_1 was notably predominantly found (number of isolates $\geq$25) in Russia, Western and Southern Europe, the whole African continent (North, Middle, West, Austral, and East), the Middle-East (Western Asia), Southern Asia, North and South America, with proportions between 43% and 99% (Figure 1). (ii) BOV_2 sub-lineage was predominantly found in Northern Europe (84% of clinical isolates), Eastern Asia (68%), Australasia (54%), South America (35%), North America (25%), Central America (9%), and Austral Africa (8%) (Figure 1). (iii) BOV_3 sub-lineage was scarcely found in our study, being limited to East Asia (24%), North America (12%), and South America (4%). (iv) $BOV\_4-CAPRAE$ lineage was predominantly found in Eastern Europe (representing 83% of isolates). This lineage represented 8% of clinical isolates in Western Europe and South America, and 6% of the isolates in North Africa. (v) BOV sub-lineage was predominantly found in Central America (59% of isolates), followed by East Africa (46% of strains); and with proportions from 12% to 23% in North, West, and Central Africa; North and South America; Western and Southern Europe; and Western and Southern Asia (Figure 1).

The database provided information on strains belonging to human hosts (n=473 isolates) and other mammalian hosts (mainly cattle; n=18769 isolates). Most of the bTB isolates from animals were found in Southern Europe (predominantly in Spain), whereas available isolates from humans were mainly found in Western Europe (n=149), Northern Europe (n=120), followed by Northern Asia or Russia (n=108) (Figure 1). Among human isolates, gender of patients was known for 301 isolates, and the global male/female sex ratio was: 173/128 = 1.35. In countries where at least 10 human isolates were recorded, the highest sex ratio was observed in Southern Europe (male/female sex ratio=3.09), as opposed to the lowest ratio in Western Europe (male/female sex ratio=0.94). Regarding the distribution of lineages between human and animal hosts (Supplementary File 1/Supplementary Figure S1), BOV_1 sub-lineage was globally more common among human hosts (n=388/473; 82.03%), followed by BOV sub-lineage (n=72/473; 15.22%). Considering the diverse sub-regions, BOV sub- lineage that predominated among animal isolates in Central America (around 88% of isolates), was also largely present among patients from Northern Europe (around 36% of isolates). Lastly, the BOV_3 sub-lineage was merely visible in patients from Eastern Asia (n=2) and South America (n=1). Table 1 shows the distribution of predominant spoligotypes in SITVITBovis.

Globally, the four genotyping methods (spoligotyping, 5-locus ETRs, 12- and 15-loci MIRU-VNTRs) showed good discriminatory power (HGDI in range of 0.930 to 0.979; Table 2). Regarding the individual VNTRs loci, we noted that QUB-11b (VNTR2163), ETR-A (VNTR2165), MIRU16 (VNTR1644), and MIRU04 (VNTR0580) displayed reasonable discriminatory power with HGDI>0.6 (Table 2).

Spoligoforest trees (Supplementary File 2) were drawn to highlight diversity of main spoligotypes (represented by a SB number or a SIT) in Africa (n=965), The Americas (n=1662), Asia (n=289), Australasia (n=105), and Europe (n=22701). In these trees, we can visualize the main spoligotypes in each continent. One may notice that, according to our database, SB0121/SIT481 was more predominant in Europe (n=5083 isolates), followed by SB0134/SIT665 (n=2021), and SB0140/SIT683 (n=1696); while SB0140/SIT683 (n=42) predominated in Australasia, followed by SB0130/SIT691 (n=31);

SB0120/SIT482 was more prevalent in Asia (n=179), followed by SB0140/SIT683 (n=85), and SB0130/SIT691 (n=32); SB0140/SIT683 was more predominant in the Americas (n=413), followed by SB0121/SIT481 (n=154), and SB0130/SIT691 (n=128); and finally, SB0944/SIT1037 was more predominant in Africa (n=136), followed by SB0121/SIT481 (n=99), and SB1176/SIT3453 (n=59). Besides, a supplementary file was added in order to allow comparisons between different regions (Supplementary File 3).

Proposal of an international consensus schema based on MIRU-VNTRs loci

Several studies have reported affordable and suitable sets of VNTRs loci to discriminate bTB isolates [31, 32, 33]. However, most of these studies have been based on specific countries. In SITVITBovis database, we have gathered data from various countries, providing a wider view of bTB diversity. According to pooled data in our database, an optimal consensus set could be based on 13 MIRU-VNTRs loci showing reasonably high discriminatory power (HGDI>0.4), i.e.: QUB-11b (VNTR2163), ETR-A (VNTR2165), MIRU16 (VNTR1644), MIRU04 (VNTR0580), ETR-B (VNTR2461), QUB-4156 (VNTR4156), MIRU31 (VNTR3192), MIRU10 (VNTR0960), ETR-C (VNTR0577), QUB-26 (VNTR4052), Mtub21 (VNTR1955), Mtub04 (VNTR0424), Mtub30 (VNTR2401) (Table 2).

In addition to the aforementioned VNTRs, we also recommend to include QUB3232 (VNTR3232) as suggested earlier by other studies [31, 32, 34], to further increase the discriminatory power in bTB diversity analyses.

Distribution of patient age groups

Comparison with SITVIT2 database showed that the distribution of patient age groups of *M. bovis* isolates (belonging to BOV_1 sublineage) was different from age group distributions in patients infected with other MTBC members (p-value<0.0001). When comparing the distribution of age groups 0-20 years, 21-40 years, 41-60 years and >60 years among patient isolates belonging to H3, LAM9, T1, Beijing, CAS1-Delhi and EAI5 sublineages, we noticed that patient isolates belonging to BOV_1 sublineage were more common among younger (age group 0-20 years), as well as older patients (>60 years) (Table 3).

Considering only the bTB strains isolated in Europe (north, south, and west – with most of the available data), we noted a significant disparity in the distribution of age groups (p-value<0.0001). For all age groups (0-20 years, 21-40 years, 41-60 years, and >60 years), we observed that the proportion of patients in Western Europe (EURO-W) was similar in each age group, representing around 20% (from 18 to 24%) of cases, except for the age group >60 years which represented about 35% of patients (Supplementary File 1/Supplementary Figure S2A). On the contrary, a majority of patients in Southern Europe (EURO-S) belonged to the age group 21-40 years (51% of patients), as opposed to the age group >60 years (81% of cases) in Northern Europe (EURO-N). These observations suggest that bovine tuberculosis is a major problem primarily affecting the working population in Southern Europe, while it rather concerns reactivation cases among senior patients in Northern Europe.

Discussion

Results obtained from this study suggested an important heterogeneity in worldwide distribution of bTB isolates. Both significant cleavages and similarities were observed in the geographical distribution of various bTB sublineages defined in our database, as well as for the various bTB genotypes.

Diversity, presence and/or absence of certain spoligotyping spacers have been revealed as being particularly useful for discriminating specific bTB isolates circulating worldwide [16, 13]. Our web based tool allows to have a global overview of bTB genotypes (including spoligotypes, E-locus ETRs, 12- and 15-loci MIRU-VNTRs). By using SITVITBovis tool, users can remarkably visualize the geographical position (at sub-region, country, or city level) of specific genotypes in addition to available epidemiological data as well as drug resistance information; the latter being an important health issue in various regions of the world [35].

MIRU-VNTR method obviously increases the discriminatory power of spoligotyping, nonetheless spoligotyping alone is still a reliable method to differentiate *M. bovis* isolates (Table 2). Several studies have focused on the diversity of MIRU-VNTRs and/or spoligotypes involved in bTB [31, 32]. However, the majority of these studies generally focused on limited geographical areas.

In our analysis, spoligotyping was globally considered as a reasonable typing method to discriminate bTB isolates (with a global HGDI

of 0.930). However, according to recent research, its discriminatory power could vary in function of countries [33]. Despite an international consensus scheme of using MIRU-VNTRs loci concomitantly with spoligotyping for improved characterization of MTBC [4, 5, 6, 15], only selected groups working on bTB isolates have used such an approach [31, 32, 33, 34]. Henceforth, the scarcity of MIRU-VNTR data in SITVITBovis should be improved by adding new data in future updates. Nonetheless, with data on 25,741 isolates (75 countries of isolation corresponding to 60 countries of origin), SITVITBovis still incorporates valuable data from other resources. Unfortunately, information on host is not always provided for all collected bTB isolates in published literature, which hampers efforts to precisely calculate the real incidence of bTB among human vs. other animal hosts. Due to the lack of available information, we did not address the issues of clonal complexes of *M. bovis* marked by specific regions of deletions (RDs) or single nucleotide polymorphisms (SNPs) in the present study. Nevertheless, information on RDs may be obtained by querying the database and exporting results into an Excel file. Last but not least, WGS and SNPs are being frequently used today to decipher bTB transmission and identify lineages involved [36, 37]. Studies using WGS already provide significant insights in bTB diversity, distribution and evolution [10, 11, 12], and may help identify hosts that could serve as reservoir of bTB infection and spread. As an illustration, a preliminary table linking WGS and classical genotyping data for some bTB isolates is available (Supplementary Table S3).

As the genomic information becomes more easily accessible in conjunction with geographical origin and hosts, we plan to automatically extract from sequencing data a number of variables such as specific phylogenetic markers, drug resistance, genomic determinants of virulence/pathogenicity, or other relevant information. This novel generation of database(s) will be able to link the newly generated information with the existing data on classical genotyping information vs. hosts, and geographical mapping. The development of all these tools in future bTB genotyping databases would possibly allow anticipating bTB outbreaks among humans and animal hosts alike.

Conclusion and Perspectives

In summary, our study provides a global overview of bTB distribution, highlighting potential relationships between bTB genotypes and affected hosts as well as other epidemiological features. SITVITBovis, with its correlation with other existing resources (such as Mbovis.org), provides a tentative picture of bTB circulation in the world, and therefore represents an non-negligible resource for monitoring bovine tuberculosis, and make correlations between epidemiological, macro-geographical and other data available.

Future developments aim to enrich the database with WGS data in conjunction with geographical origin and hosts. Development of automated extraction of knowledge related to specific phylogenetic markers, drug resistance, genomic determinants of virulence/pathogenicity, or other relevant information will soon make it possible to link the newly generated information with the existing data generated using classical genotyping tools. The development of such a strategy in conjunction with information on hosts, and subsequent geographical mapping will boost our efforts to generate a real comprehensive snapshot of global bTB diversity. Helpful to better identify, treat and control bTB, such a tool will be highly beneficial for both medical and veterinarian specialists, as well as public health authorities.

Competing interests

There is NO Competing Interest.

Author contributions statement

N.R. designed and supervised the study. D.C., I.C.M., and A.D. deevloped the database, and designed the algorithms allowing to query the web application. Y.R. helped in the analysis of WGS data. D.C. and N.R. wrote and reviewed the manuscript.

Acknowledgments

We thank more than 300 investigators who provided M. bovis data for SpolDB4, SITVITWEB, and SITVIT2 databases. We are highly grateful to Igor Mokrousov, Thierry Zozio, Julie Millet, Véronique Hill, and Elisabeth Streit for helpful discussions. DC was awarded a Ph.D. fellowship by the European Social Funds through the Regional Council of Guadeloupe.

Funding

The work done at Institut Pasteur de la Guadeloupe was supported by a FEDER grant, financed by the European Union and Guadeloupe Region (Programme Opérationnel FEDER-Guadeloupe-Conseil Régional 2014-2020, Grant number 2015-FED-192).

References

1. World Health Organization (WHO) Global tuberculosis report 2020. (2020) https://www.who.int/publications/i/item/9789240013131 (accessed June, 7th, 2021)
2. Smith, N.H., Hewinson, R.G., Kremer, K., Brosch, R., Gordon, S.V.: Myths and misconceptions: the origin and evolution of Mycobacterium tuberculosis. Nat Rev Microbiol **7** (2009) 537–544.
3. Kamerbeek, J., Schouls, L., Kolk, A., van Agterveld, M., van Soolingen, D., Kuijper, S., Bunschoten, A., Molhuizen, H., Shaw, R., Goyal, M., van Embden, J.: Simultaneous detection and strain differentiation of Mycobacterium tuberculosis for diagnosis and epidemiology. J Clin Microbiol **35** (1997) 907–914.
4. Supply, P., Allix, C., Lesjean, S., Cardoso-Oelemann, M., Rusch-Gerdes, S., Willery, E., Savine, E., de Haas, P., van Deutekom, H., Roring, S., Bifani, P., Kurepina, N., Kreiswirth, B., Sola, C., Rastogi, N., Vatin, V., Gutierrez, M.C., Fauville, M., Niemann, S., Skuce, R., Kremer, K., Locht, C., van Soolingen, D.: Proposal for Standardization of Optimized Mycobacterial Interspersed Repetitive Unit-Variable-Number Tandem Repeat Typing of Mycobacterium tuberculosis. J. Clin. Microbiol **44** (2006) 4498—4510.
5. Rastogi, N., Sola, C.: Chapter 2 – Molecular evolution of the Mycobacterium tuberculosis complex. In Tuberculosis 2007: from basic science to patient care, Edited by Palomino JC, Leao S, Ritacco V.. Amedeo Online Textbooks **2007** (2007) 53–91.
6. García de Viedma, D., Mokrousov, I., Rastogi, N.: Innovations in the molecular epidemiology of tuberculosis. Enferm Infecc Microbiol Clin **29** (2011) 8–13.
7. Demay, C., Liens, B., Burguière, T., Hill, V., Couvin, D., Millet, J., Mokrousov, I., Sola, C., Zozio, T., Rastogi, N.: SITVITWEB – A publicly available international multimarker database for studying Mycobacterium tuberculosis genetic diversity and molecular epidemiology. Infect. Genet. Evol **12** (2012) 755-–766.
8. Comas, I., Homolka, S., Niemann, S., Gagneux, S.: Genotyping of genetically monomorphic bacteria: DNA sequencing in Mycobacterium tuberculosis highlights the limitations of current methodologies. PLoS One **4** (2009) e7815.
9. Pérez-Lago, L., Navarro, Y., García-de-Viedma, D.: Current knowledge and pending challenges in zoonosis caused by Mycobacterium bovis: A review. Res Vet Sci **97** (2013) S94–S100.
10. Lasserre, M., Fresia, P., Greif, G., Iraola, G., Castro-Ramos, M., Juambeltz, A., Nuñez, Á., Naya, H., Robello, C., Berná, L.: Whole genome sequencing of the monomorphic pathogen Mycobacterium bovis reveals local differentiation of cattle clinical isolates. BMC Genomics **19** (2018) 2.
11. Zimpel, C.K., Patané, J.S.L., Guedes, A.C.P., de Souza, R.F., Silva-Pereira, T.T., Camargo, N.C.S., de Souza Filho, A.F., Ikuta, C.Y., Neto, J.S.F., Setubal, J.C., Heinemann, M.B., Guimaraes, A.M.S.: Global Distribution and Evolution of Mycobacterium bovis Lineages. Front Microbiol **11** (2020) 843.
12. Rodrigues, R.A., Ribeiro Araújo, F., Rivera Dávila, A.M., Etges, R.N., Parkhill, J., van Tonder, A.J.: Genomic and temporal analyses of Mycobacterium bovis in southern Brazil. Microbial Genomics **7** (2021) 5.
13. Smith, N.H., Upton, P.: Naming spoligotype patterns for the RD9-deleted lineage of the Mycobacterium tuberculosis complex; www.Mbovis.org. Infect Genet Evol **12** (2012) 873—876.
14. Rodriguez-Campos, S., Schürch, A.C., Dale, J., Lohan, A.J., Cunha, M.V., Botelho, A., De Cruz, K., Boschiroli, M.L., Boniotti, M.B., Pacciarini, M., Garcia-Pelayo, M.C., Romero, B., de Juan, L., Domínguez, L., Gordon, S.V., van Soolingen, D., Loftus, B., Berg, S., Hewinson, R.G., Aranaz, A., Smith, N.H.: European 2–a clonal complex of Mycobacterium bovis dominant in the Iberian Peninsula. Infect Genet Evol **12** (2012) 866–872.
15. Couvin, D., David, A., Zozio, T., Rastogi, N.: Macro-geographical specificities of the prevailing tuberculosis epidemic as seen through SITVIT2, an updated version of the Mycobacterium tuberculosis genotyping database. Infection, Genetics and Evolution **72** (2019) 31–43
16. Rodriguez-Campos, S., González, S., de Juan, L., Romero, B., Bezos, J., Casal, C., Álvarez, J., Fernández-de-Mera, I.G., Castellanos, E., Mateos, A., Sáez-Llorente, J.L., Domínguez, L., Aranaz, A.; Spanish Network on Surveillance Monitoring of Animal Tuberculosis.: A database for animal tuberculosis (mycoDB.es) within the context of the Spanish national programme for eradication of bovine tuberculosis. Infect Genet Evol **12** (2012) 877–882.
17. Smith, N.H., Berg, S., Dale, J., Allen, A., Rodriguez, S., Romero, B., Matos, F., Ghebremichael, S., Karoui, C., Donati, C., Machado Ada, C., Mucavele, C., Kazwala, R.R., Hilty, M., Cadmus, S., Ngandolo, B.N., Habtamu, M., Oloya, J., Muller, A., Milian-Suazo, F., Andrievskaia, O., Projahn, M., Barandiarán, S.,

Macías, A., Müller, B., Zanini, M.S., Ikuta, C.Y., Rodriguez, C.A., Pinheiro, S.R., Figueroa, A., Cho, S.N., Mosavari, N., Chuang, P.C., Jou, R., Zinsstag, J., van Soolingen, D., Costello, E., Aseffa, A., Proaño-Perez, F., Portaels, F., Rigouts, L., Cataldi, A.A., Collins, D.M., Boschiroli, M.L., Hewinson, R.G., Ferreira Neto, J.S., Surujballi, O., Tadyon, K., Botelho, A., Zárraga, A.M., Buller, N., Skuce, R., Michel, A., Aranaz, A., Gordon, S.V., Jeon, B.Y., Källenius, G., Niemann, S., Boniotti, M.B., van Helden, P.D., Harris, B., Zumárraga, M.J., Kremer, K.: European 1: a globally important clonal complex of Mycobacterium bovis. Infect Genet Evol **11** (2011) 1340–1351.

18. Müller, B., Hilty, M., Berg, S., Garcia-Pelayo, M.C., Dale, J., Boschiroli, M.L., Cadmus, S., Ngandolo, B.N., Godreuil, S., Diguimbaye-Djaibé, C., Kazwala, R., Bonfoh, B., Njanpop-Lafourcade, B.M., Sahraoui, N., Guetarni, D., Aseffa, A., Mekonnen, M.H., Razanamparany, V.R., Ramarokoto, H., Djønne, B., Oloya, J., Machado, A., Mucavele, C., Skjerve, E., Portaels, F., Rigouts, L., Michel, A., Müller, A., Källenius, G., van Helden, P.D., Hewinson, R.G., Zinsstag, J., Gordon, S.V., Smith, N.H.: African 1, an epidemiologically important clonal complex of Mycobacterium bovis dominant in Mali, Nigeria, Cameroon, and Chad. J Bacteriol **191** (2009) 1951–1960.
19. Berg, S., Garcia-Pelayo, M.C., Müller, B., Hailu, E., Asiimwe, B., Kremer, K., Dale, J., Boniotti, M.B., Rodriguez, S., Hilty, M., Rigouts, L., Firdessa, R., Machado, A., Mucavele, C., Ngandolo, B.N., Bruchfeld, J., Boschiroli, L., Müller, A., Sahraoui, N., Pacciarini, M., Cadmus, S., Joloba, M., van Soolingen, D., Michel, A.L., Djønne, B., Aranaz, A., Zinsstag, J., van Helden, P., Portaels, F., Kazwala, R., Källenius, G., Hewinson, R.G., Aseffa, A., Gordon, S.V., Smith, N.H.: African 2, a clonal complex of Mycobacterium bovis epidemiologically important in East Africa. J Bacteriol **193** (2011) 670–678.
20. Aranaz, A., Liébana, E., Mateos, A., Dominguez, L., Vidal, D., Domingo, M., Gonzolez, O., Rodriguez-Ferri, E.F., Bunschoten, A.E., Van Embden, J.D., Cousins, D.: Spacer oligonucleotide typing of Mycobacterium bovis strains from cattle and other animals: a tool for studying epidemiology of tuberculosis. J Clin Microbiol **34** (1996) 2734–2740.
21. Frothingham, R., Meeker-O'Connell, W.A.: Genetic diversity in the Mycobacterium tuberculosis complex based on variable numbers of tandem DNA repeats. Microbiology **144** (1998) 1189–1196.
22. Xia, E., Teo, Y.Y., Ong, R.T.H.: SpoTyping: fast and accurate in silico Mycobacterium spoligotyping from sequence reads. Genome Med **8** (2016) 19.
23. Tang, C.Y., Ong, R.T.: MIRUReader: MIRU-VNTR typing directly from long sequencing reads. Bioinformatics **36** (2020) 1625–1626.
24. Bankevich, A., Nurk, S., Antipov, D., Gurevich, A.A., Dvorkin, M., Kulikov, A.S., Lesin, V.M., Nikolenko, S.I., Pham, S., Prjibelski, A.D., Pyshkin, A.V., Sirotkin, A.V., Vyahhi, N., Tesler, G., Alekseyev, M.A., Pevzner, P.A.: SPAdes: a new genome assembly algorithm and its applications to single-cell sequencing. J Comput Biol **19** (2012) 455–477.
25. Couvin, D., Segretier, W., Stattner, E., Rastogi, N.: Novel methods included in SpolLineages tool for fast and precise prediction of Mycobacterium tuberculosis complex spoligotype families. Database (Oxford) **2020** (2020) baaa108.
26. Phelan, J., O'Sullivan, D.M., Machado, D., Ramos, J., Oppong, Y.E.A., Campino, S., O'Grady, J., McNerney, R., Hibberd, M.L., Viveiros, M., Huggett, J.F., Clark, T.G.: Integrating informatics tools and portable sequencing technology for rapid detection of resistance to anti-tuberculous drugs. Genome Med **11** (2019) 41.
27. Freschi, L., Vargas Jr, R., Hussain, A., Mostofa Kamal, S.M., Skrahina, A., Tahseen, S., Ismail, N., Barbova, A., Niemann, S., Maria Cirillo, D., Dean A.S., Zignol M., Farhat M.R.: Population structure, biogeography and transmissibility of Mycobacterium tuberculosis. bioRxiv (2020)
28. Tang, C., Reyes, J.F., Luciani, F., Francis, A.R., Tanaka, M.M.: SpolTools: online utilities for analyzing spoligotypes of the Mycobacterium tuberculosis complex. Bioinformatics **24** (2008) 2414–2415.
29. Reyes, J.F., Francis, A.R., Tanaka, M.M.: Models of deletion for visualizing bacterial variation: an application to tuberculosis spoligotypes. BMC Bioinformatics **9** (2008) 496.
30. Hunter, P.R., Gaston, M.A.: Numerical index of the discriminatory ability of typing systems: an application of Simpson's index of diversity. J Clin Microbiol **26** (1988) 2465–2466.
31. Duarte, E.L., Domingos, M., Amado, A., Cunha, M.V., Botelho, A.: MIRU-VNTR typing adds discriminatory value to groups of Mycobacterium bovis and Mycobacterium caprae strains defined by spoligotyping. Vet Microbiol **143** (2010) 299–306.
32. Rodriguez-Campos, S., Navarro, Y., Romero, B., de Juan, L., Bezos, J., Mateos, A., Golby, P., Smith, N.H., Hewinson, G.R., Domínguez, L., García-de-Viedma, D., Aranaz, A.: Splitting of a prevalent Mycobacterium bovis spoligotype by variable-number tandem-repeat typing reveals high heterogeneity in an evolving clonal group. J Clin Microbiol **51** (2013) 3658–3665.
33. Navarro, Y., Herranz, M., Romero, B., Bouza, E., Domínguez, L., de Juan, L., García-de-Viedma, D.: High-throughput multiplex MIRU-VNTR typing of Mycobacterium bovis. Res Vet Sci **96** (2014) 422–425.

34. Lamine-Khemiri, H., Martínez, R., García-Jiménez, W.L., Benítez-Medina, J.M., Cortés, M., Hurtado, I., Abassi, M.S., Khazri, I., Benzarti, M., Hermoso-de-Mendoza, J.: Genotypic characterization by spoligotyping and VNTR typing of Mycobacterium bovis and Mycobacterium caprae isolates from cattle of Tunisia. Trop Anim Health Prod **46** (2014) 305–311.
35. Vazquez-Chacon, C.A., Martínez-Guarneros, A., Couvin, D., González-Y-Merchand, J.A., Rivera-Gutierrez, S., Escobar-Gutierrez, A., De-la-Cruz López, J.J., Gomez-Bustamante, A., Gonzalez-Macal, G.A., Gonçalves Rossi, L.M., Muñiz-Salazar, R., Rastogi, N., Vaughan, G.: Human multidrug-resistant Mycobacterium bovis infection in Mexico. Tuberculosis (Edinb.) **95** (2015) 802–809.
36. Kohl, T.A., Utpatel, C., Niemann, S., Moser, I.: Mycobacterium bovis Persistence in Two Different Captive Wild Animal Populations in Germany: a Longitudinal Molecular Epidemiological Study Revealing Pathogen Transmission by Whole-Genome Sequencing. J Clin Microbiol **56** (2018) e00302–18.
37. Lipworth, S., Jajou, R., de Neeling, A., Bradley, P., van der Hoek, W., Maphalala, G., Bonnet, M., Sanchez-Padilla, E., Diel, R., Niemann, S., Iqbal, Z., Smith, G., Peto, T., Crook, D., Walker, T., van Soolingen, D.: SNP-IT Tool for Identifying Subspecies and Associated Lineages of Mycobacterium tuberculosis Complex. Emerg Infect Dis **25** (2019) 482–488.
