# Supplementary material for: SITVITBovis—a publicly available database and mapping tool to get an improved overview of animal and human cases caused by Mycobacterium bovis
Source: Database (Oxford). 2022 Jan 13;2022:baab081. doi: 10.1093/database/baab081 (PMC8962452; doi:10.1093/database/baab081)
Supplement: baab081_Supp [file baab081_supp.zip › Supplementary_File_1_SITVITBovis_Supplementary_data.pdf]

Supplementary File 1

SITVITBovis - a publicly available database and mapping tool to get an improved overview of animal and human cases caused by Mycobacterium bovis.

David Couvin, Iñaki Cervera-Marzal, Audrey David, Yann Reynaud, Nalin Rastogi

1. Background information on *M. bovis*

Bovine tuberculosis (bTB) can spread to humans through inhalation of infectious droplet nuclei and by ingestion of raw milk or other dairy products (**Thoen et al., 2006**). In industrialized countries eradication programs and pasteurization of milk have significantly reduced the risk of milk-borne diseases and other infections, but reservoirs in wildlife make complete eradication difficult. Humans are spillover hosts of *M. bovis*, which is principally transmitted between animals, either wild or farmed. Therefore, the wildlife-livestock-human interface appears to be one of the central research themes to eradicate this disease (**Nugent, 2011**; **De Garine-Wichatitsky et al., 2013**), but an important lack of data blocks the comprehension of this spread. It is obvious that bTB would not reach such a high proportion among humankind if it was not transmitted by food (**Francis, 1958**). Moreover, consumption of dairy products is a general practice in many countries. bTB is also likely to occur due to trade or transportation of cattle or other possible animal reservoirs (wildlife) carrying the bacterium responsible for the disease. Strains of *M. bovis* are inherently resistant to pyrazinamide (PZA) and, in some cases, exhibit a low level of resistance to isoniazid (**Ritz et al., 2009** ; **Scorpio and Zhang, 1996**}. Some studies have also shown that *M. bovis* strains were significantly associated with extra-pulmonary TB (**Lari et al., 2009** ; **Fallico et al., 2014**).

2. Additional information

Spoligotyping patterns being predominant in wildlife

Supplementary Table S1 showed spoligotypes of MTBC species found in several animals.

<u>Supplementary Table S1</u>. spoligotypes of MTBC species found in several animals

BioProject	Accession	Species	Spoligotype Description (octal)
PRJEB40500	GCA_904810325	M. microti	□□□□□□□□□□□□□□□□□□□□□□□□□□□□□□□□□□□□■■□□□□□ (000000000000600)
PRJNA377261	GCA_002982275	M. pinnipedii	□□□■■■■□□□□□□□□□□□□□□□■■□■■■■■■□□■■■■□□□□□□ (074000033747400)
PRJNA320514	GCA_001652545	M. mungi	■■□■■■□■□■■□□□□□□□□□□□□□□□□□□□□□□□□■■□■■■■ (672600000000671)
PRJNA327952	GCA_001835535	Dassie bacillus (similar to M. suricattae)	■■■■■■□■□■■■■■■■■■■□□□□□□□□□□□□□□□■■■□■■■■ (772777400001671)
PRJNA72211	AGAK01000000	M. oryx	□□□ (000000000000000)
PRJNA316192	GCA_001648835	M. leprae	□□□ (000000000000000)

Note that BioProject and Accession numbers were collected from the European Nucleotide Archive (https://www.ebi.ac.uk/ena/browser/home).

Mbovis.org database (https://www.mbovis.org/) was used to extract the SB numbers matching with some SITs numbers from SITVITBovis database (**Supplementary Table S2**).

Supplementary Files 2 and **3** showed distribution of main spoligotypes in different geographic regions.

Supplemental Figure S1 showed the distribution of lineages between human and animal hosts.

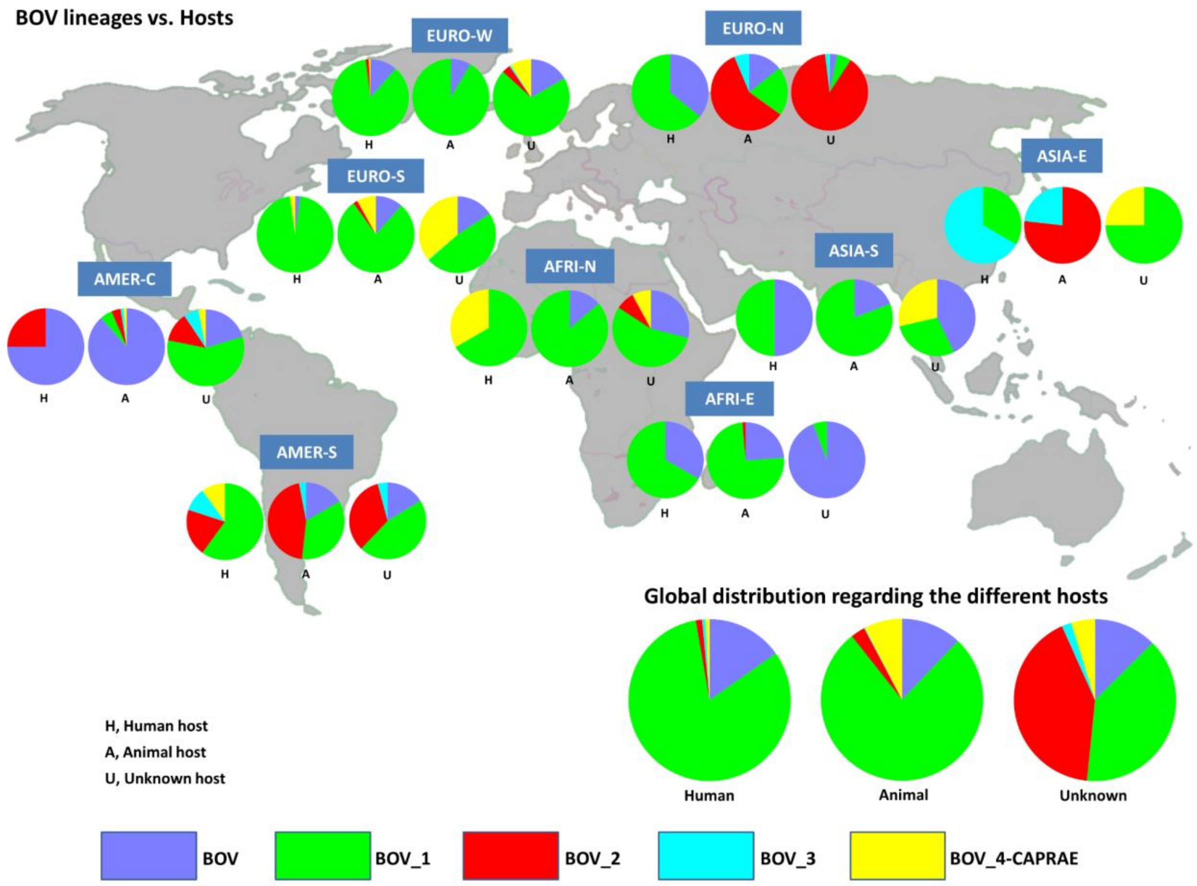

Supplemental Figure S1. Global distribution map of BOV lineages vs. hosts according to the SITVITBovis database.

Trends over time, diversity of spoligotypes, HIV serology and Drug Resistance
Basing on data collected in SITVITBovis database, the proportion of bTB in humans represented 5.18%, 6.12%, 10.47%, 27.81% and 35.82% in the years 1994, 1998, 2001, 2006, and 2009 respectively (**Supplementary Figure S2B**). However, because of the scarcity of available data, we cannot draw significant epidemiological conclusions.

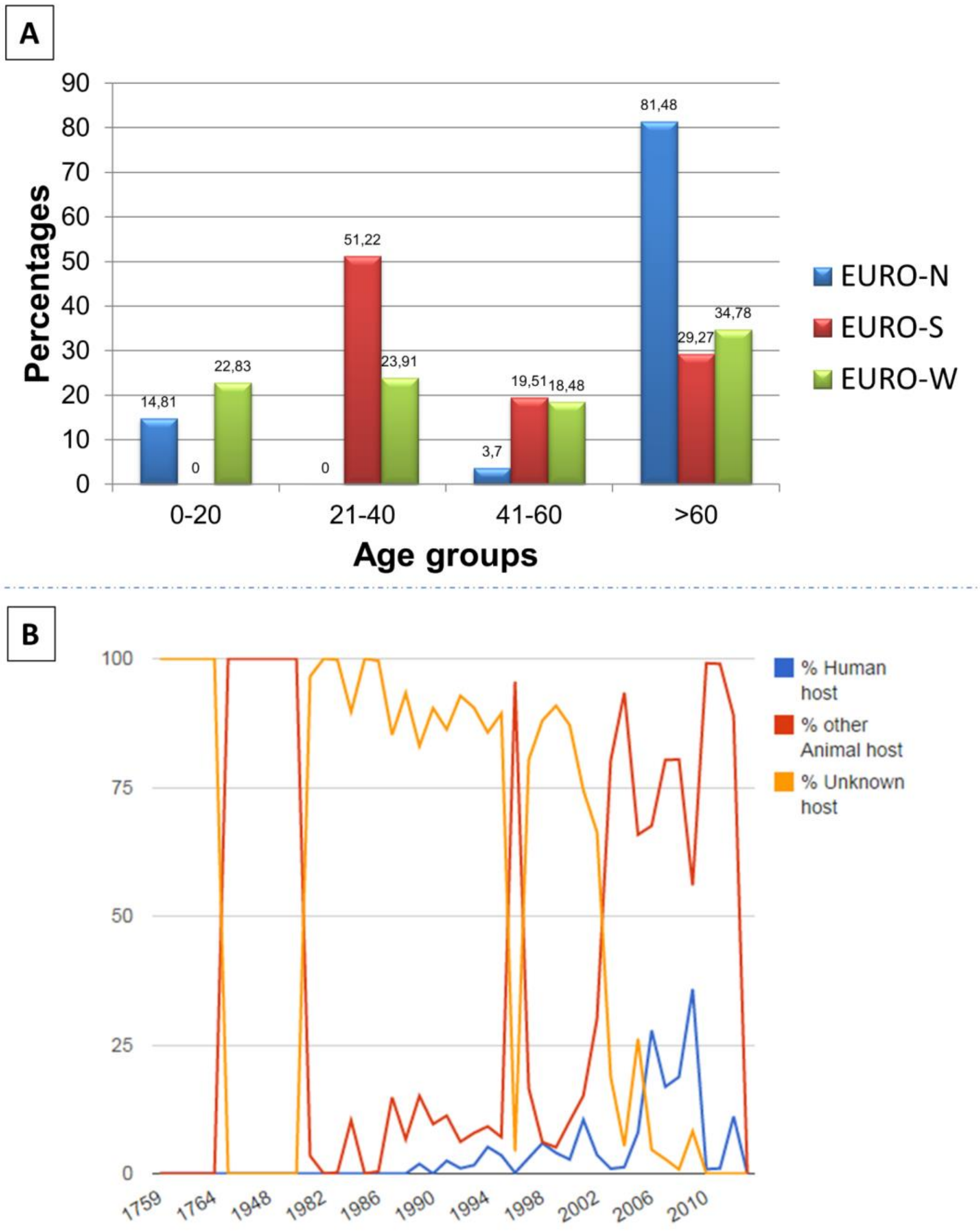

Supplementary Figure S2. (A) Distribution of age groups of bTB patients in Northern, Southern, and Western Europe; (B) Overall temporal evolution of bTB based on different hosts (humans and animals) collected in this database (http://www.pasteur-guadeloupe.fr:8081/SITVIT_Bovis/stata.jsp).

A Minimum spanning tree (MST) was drawn to differentiate spoligotypes involved in humans, cattle, and wildlife (**Supplementary Figure S3**). Some spoligotypes were shared by humans, cattle and other mammals such as SIT691/BOV_1. Predominant patterns such as SIT696/BOV_1, SIT695/BOV, SIT645/BOV_4-CAPRAE and SIT3714/BOV_4-CAPRAE, were merely present in strains isolated from cattle. Around 10% of isolates belonging to SIT683/BOV_2 were found among wildlife.

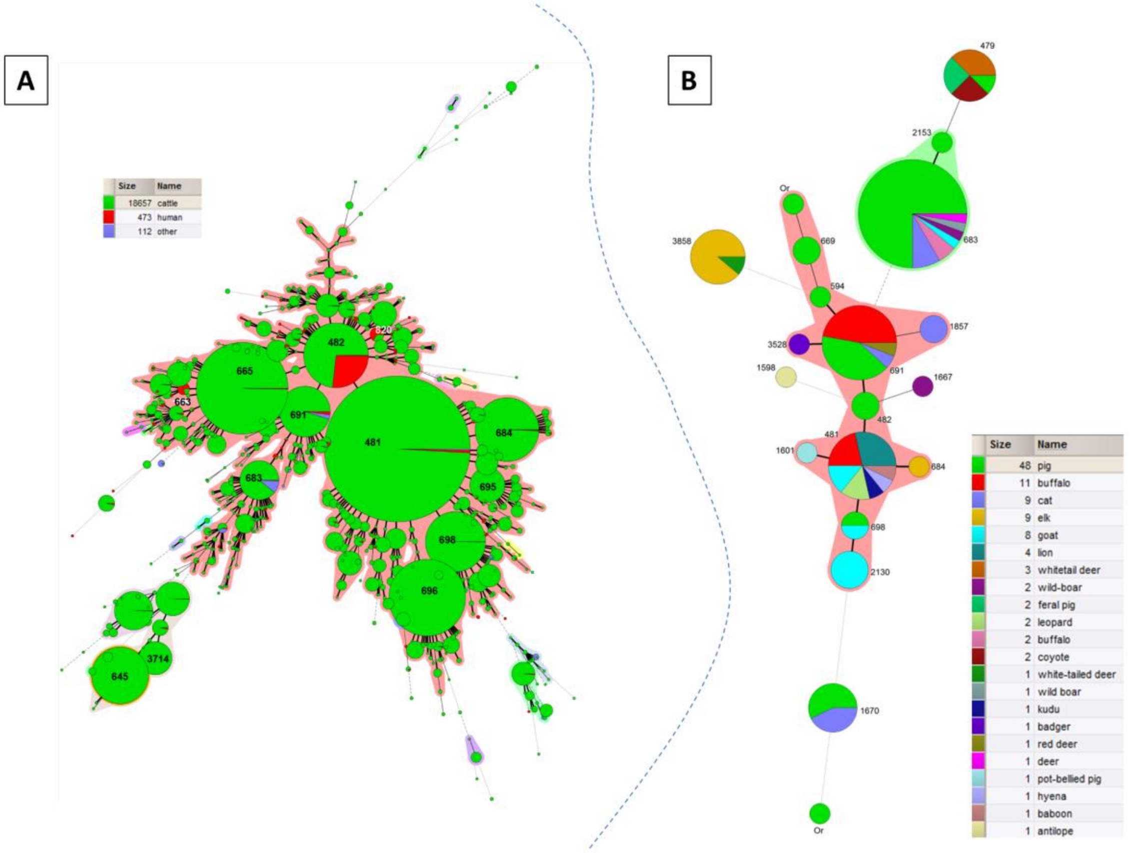

<u>Supplementary Figure S3</u>. Minimum Spanning Trees (MSTs) constructed with BioNumerics software (version 6.6), based on spoligotypes; (A) tree drawn in function of the various available hosts: human (in red; n=473), cattle (in green; n=18657), and wildlife (in blue; n=112); (B) MST constructed in function of wildlife (n=112 isolates). The size of the nodes (spoligotypes) was proportional to the number of strains shared by a given profile. The number inside or beside nodes indicates the SIT number. The shape of the links between nodes represents the distance separating each profile (dashed and dotted lines designate 3 or more changes between spoligotypes, whereas continuous gray and bolder lines designate 2 changes or less).

Supplementary Table S3 showed a preliminary table linking WGS and classical genotyping data for some bTB isolates.

Supplementary Table S4 showed global diversity (HGDI) of the 43 spacers of all spoligotypes contained in SITVITBovis. We noted that spacer 21, spacer 6, and spacer 11 displayed the highest discriminatory powers representing 0.499, 0.418, and 0.415 respectively.

Supplementary Table S4. Global diversity (HGDI) of the 43 spacers of all spoligotypes contained in SITVITBovis

43 Spoligotyping Spacers (SP)	Hunter-Gaston Diversity	95% Confidence Interval
SP21	0.499	0.499 - 0.500
SP06	0.418	0.414 - 0.423
SP11	0.415	0.410 - 0.419
SP12	0.378	0.373 - 0.383
SP08	0.369	0.364 - 0.375
SP10	0.369	0.363 - 0.374
SP04	0.361	0.356 - 0.367
SP05	0.361	0.356 - 0.367
SP28	0.273	0.267 - 0.279
SP15	0.238	0.232 - 0.244
SP13	0.209	0.203 - 0.215
SP07	0.207	0.201 - 0.213
SP14	0.195	0.189 - 0.201
SP33	0.195	0.189 - 0.201
SP30	0.186	0.180 - 0.191
SP27	0.177	0.172 - 0.183
SP29	0.177	0.171 - 0.183
SP32	0.164	0.159 - 0.170
SP01	0.161	0.155 - 0.166
SP26	0.157	0.151 - 0.162
SP31	0.156	0.150 - 0.162
SP37	0.131	0.126 - 0.136
SP02	0.107	0.102 - 0.112
SP19	0.107	0.102 - 0.112
SP23	0.100	0.095 - 0.105
SP22	0.095	0.090 - 0.100
SP34	0.082	0.077 - 0.086
SP18	0.068	0.064 - 0.072
SP17	0.066	0.061 - 0.070
SP20	0.064	0.059 - 0.068
SP35	0.050	0.046 - 0.053
SP24	0.046	0.042 - 0.049
SP36	0.045	0.042 - 0.049
SP38	0.033	0.030 - 0.036
SP25	0.017	0.015 - 0.020
SP16	0.004	0.003 - 0.005
SP09	0.000	0.000 - 0.000
SP03	0.000	0.000 - 0.000
SP40	0.000	0.000 - 0.000
SP42	0.000	0.000 - 0.000
SP39	0.000	0.000 - 0.000
SP41	0.000	0.000 - 0.000
SP43	0.000	0.000 - 0.000

Information concerning HIV-serology was available for 19 isolates distributed in Spain (n=17 isolates, including 14 HIV-positive and 3 HIV-negative), in Peru (n=1 isolate from a HIV-positive patient), and in Italy (n=1 isolate from a HIV-negative patient). Among bTB strains isolated from human host (n=473), information about drug resistance was unknown for n=365 (77.17% of isolates) and known for n=108 (i.e. 22.83% of isolates). Among the known drug resistance information, (i) Code 1 (Pansusceptible) accounted for n=33 (30.56%) isolates, (ii) Code 2 (MDR-TB) accounted for n=2 (1.85%) isolates, (iii) Code 3 (any drug resistant TB) accounted for n=46 (42.59%) isolates, and (iv) Code 4 (XDR-TB) accounted for n=27 (25%) isolates (all 27 XDR strains have the same spoligotype (SIT663/BOV_1) and were isolated in Spain).

Supplementary Figure S4 provides a global overview of distribution and evolution of bovine sublineages over time and/or sub-region. The global proportion of BOV sublineage was 20% between 1990 and 2005, and this proportion reached 27.16% of isolates in 2005-2012.

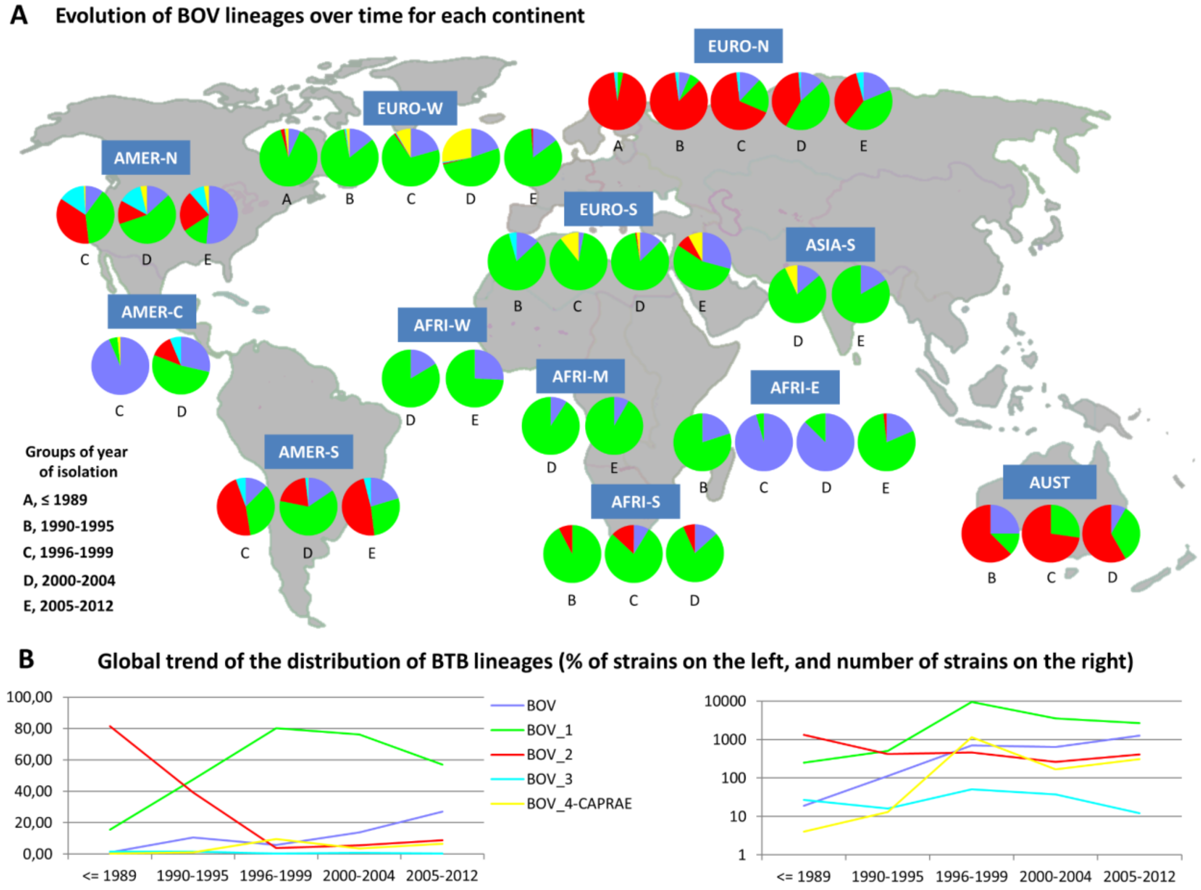

Supplementary Figure S4. (A) Global map showing evolution of BOV lineages over time; (B) Percentage evolution of BOV lineages over time.

Supplementary Table S5 provides information on the country of origin of bTB isolates, for a total of 68 isolates. This table highlights potential paths of transmission of bTB between countries. One may notice that several recorded bTB isolates were from Morocco (n=20), and their destinations or countries of isolation mainly concerned European countries

(Belgium n=5, Denmark n=1, Spain n=3, France n=2, Italy n=7), however 2 Moroccan isolates were observed in Tunisia. Nevertheless, we also noticed an important number of strains from France (n=13) isolated in Tunisia. However, the information available concerning the origin of isolates was insufficient to draw significant conclusions.

Supplementary Table S5. Information on the country of origin and the country of isolation of bTB strains.

**Country of Origin (Iso alpha 2 code)**	**Country of Isolation (Iso alpha 2 code)**	**Number of isolates**
Australia (AU)	Tunisia (TN)	**3**
Burkina Faso (BF)	Italy (IT)	**1**
Burundi (BI)	Belgium (BE)	**1**
Republic of the Congo (CG)	France (FR)	**1**
Cameroon (CM)	Spain (ES)	**1**
Djibouti (DJ)	France (FR)	**1**
Denmark (DK)	Tunisia (TN)	**2**
Algeria (DZ)	France (FR)	**2**
Algeria (DZ)	Tunisia (TN)	**1**
Spain (ES)	Tunisia (TN)	**1**
France (FR)	Tunisia (TN)	**13**
Ghana (GH)	Spain (ES)	**1**
Greenland (GL)	Denmark (DK)	**1**
Iran (IR)	Denmark (DK)	**1**
Cambodia (KH)	United States (US)	**1**
Morocco (MA)	Belgium (BE)	**5**
Morocco (MA)	Denmark (DK)	**1**
Morocco (MA)	Spain (ES)	**3**
Morocco (MA)	France (FR)	**2**
Morocco (MA)	Italy (IT)	**7**
Morocco (MA)	Tunisia (TN)	**2**
Mexico (MX)	United States (US)	**3**
Netherlands (NL)	Argentina (AR)	**1**
Peru (PE)	Italy (IT)	**1**
Philippines (PH)	Italy (IT)	**1**
Sweden (SE)	Tunisia (TN)	**1**
Tunisia (TN)	France (FR)	**1**
Tunisia (TN)	Italy (IT)	**1**
Turkey (TR)	Denmark (DK)	**1**
Turkey (TR)	France (FR)	**2**
Tanzania (TZ)	Denmark (DK)	**1**
United States (US)	Norway (NO)	**1**
United States (US)	Tunisia (TN)	**2**
Vietnam (VN)	Denmark (DK)	**1**

References:

Thoen, C., Lobue, P., de Kantor, I.: The importance of *Mycobacterium bovis* as a zoonosis. Vet Microbiol 112 (2006) 339-345.

Nugent, G.: Maintenance, spillover and spillback transmission of bovine tuberculosis in multi-host wildlife complexes: a New Zealand case study. Vet Microbiol 151 (2011) 34-42.

De Garine-Wichatitsky, M., Caron, A., Kock, R., Tschopp, R., Munyeme, M., Hofmeyr, M., Michel, A.: A review of bovine tuberculosis at the wildlifelivestock-human interface in sub-Saharan Africa. Epidemiol Infect 141 (2013) 1342-1356.

Francis, J.: Tuberculosis in animals and man. Cassell and Company, London, UK (1958) 357pp.

Ritz, N., Tebruegge, M., Connell, T.G., Sievers, A., Robins-Browne, R., Curtis, N.: Susceptibility of *Mycobacterium bovis* BCG vaccine strains to antituberculous antibiotics. Antimicrob Agents Chemother 53 (2009) 316-318.

Scorpio, A., Zhang, Y.: Mutations in pncA, a gene encoding pyrazinamidase/nicotinamidase, cause resistance to the antituberculous drug pyrazinamide in tubercle bacillus. Nat Med 2 (1996) 662-667.

Lari, N., Rindi, L., Cristofani, R., Rastogi, N., Tortoli, E., Garzelli, C.: Association of *Mycobacterium tuberculosis* complex isolates of BOVIS and Central Asian (CAS) genotypic lineages with extrapulmonary disease. Clin Microbiol Infect 15 (2009) 538-543.

Fallico, L., Couvin, D., Peracchi, M., Pascarella, M., Franchin, E., Lavezzo, E., Rassu, M., Manganelli, R., Rastogi, N., Pal, G.: Four year longitudinal study of *Mycobacterium tuberculosis* complex isolates in a region of North-Eastern Italy. Infect Genet Evol 26 (2014) 58-64.
