# Supplementary figures and images for: SITVITBovis—a publicly available database and mapping tool to get an improved overview of animal and human cases caused by Mycobacterium bovis
Source: Database (Oxford). 2022 Jan 13;2022:baab081. doi: 10.1093/database/baab081 (PMC8962452; doi:10.1093/database/baab081)

## Supplementary File 2

**AFRICA (n=965)**

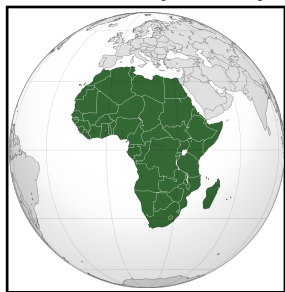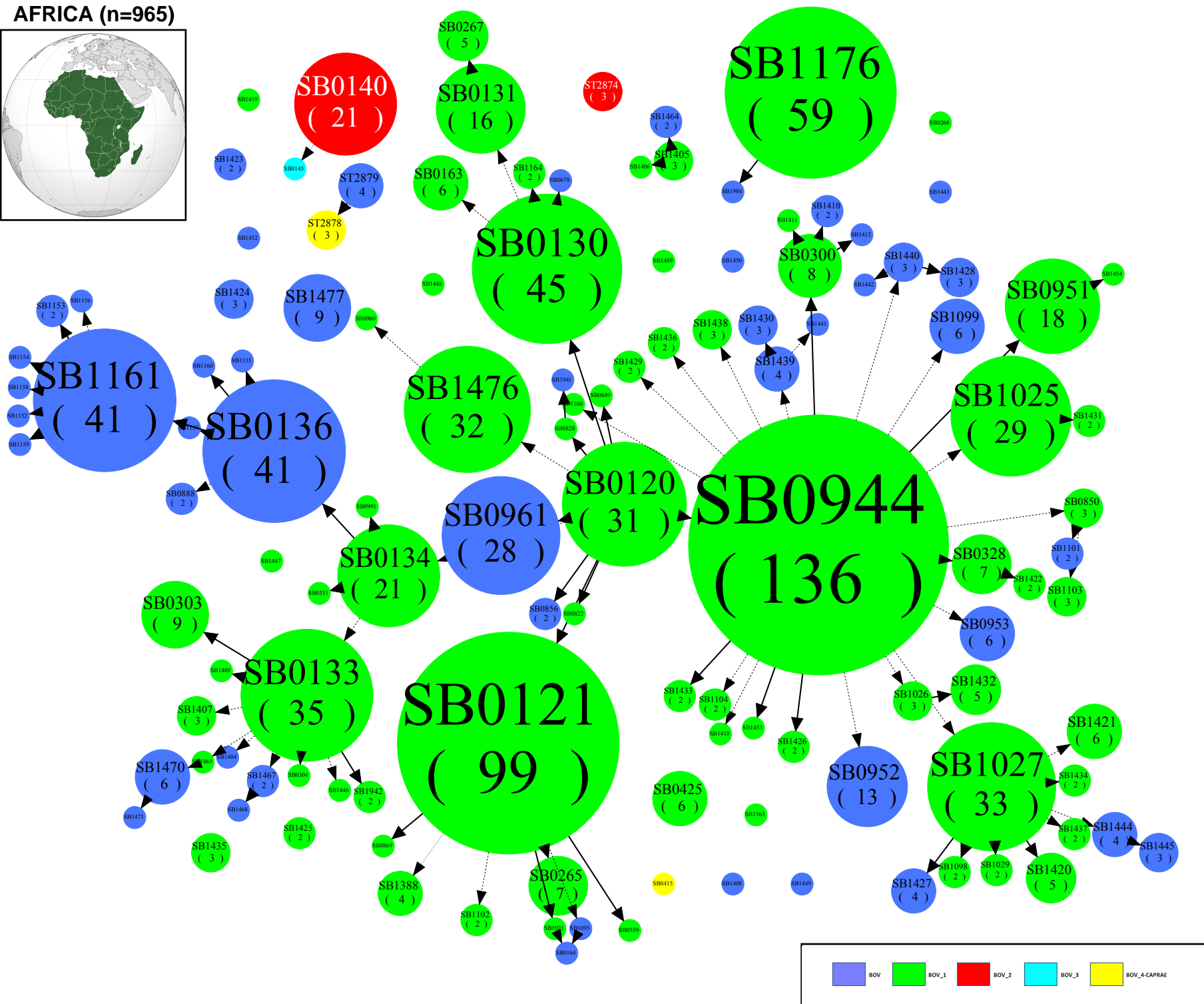

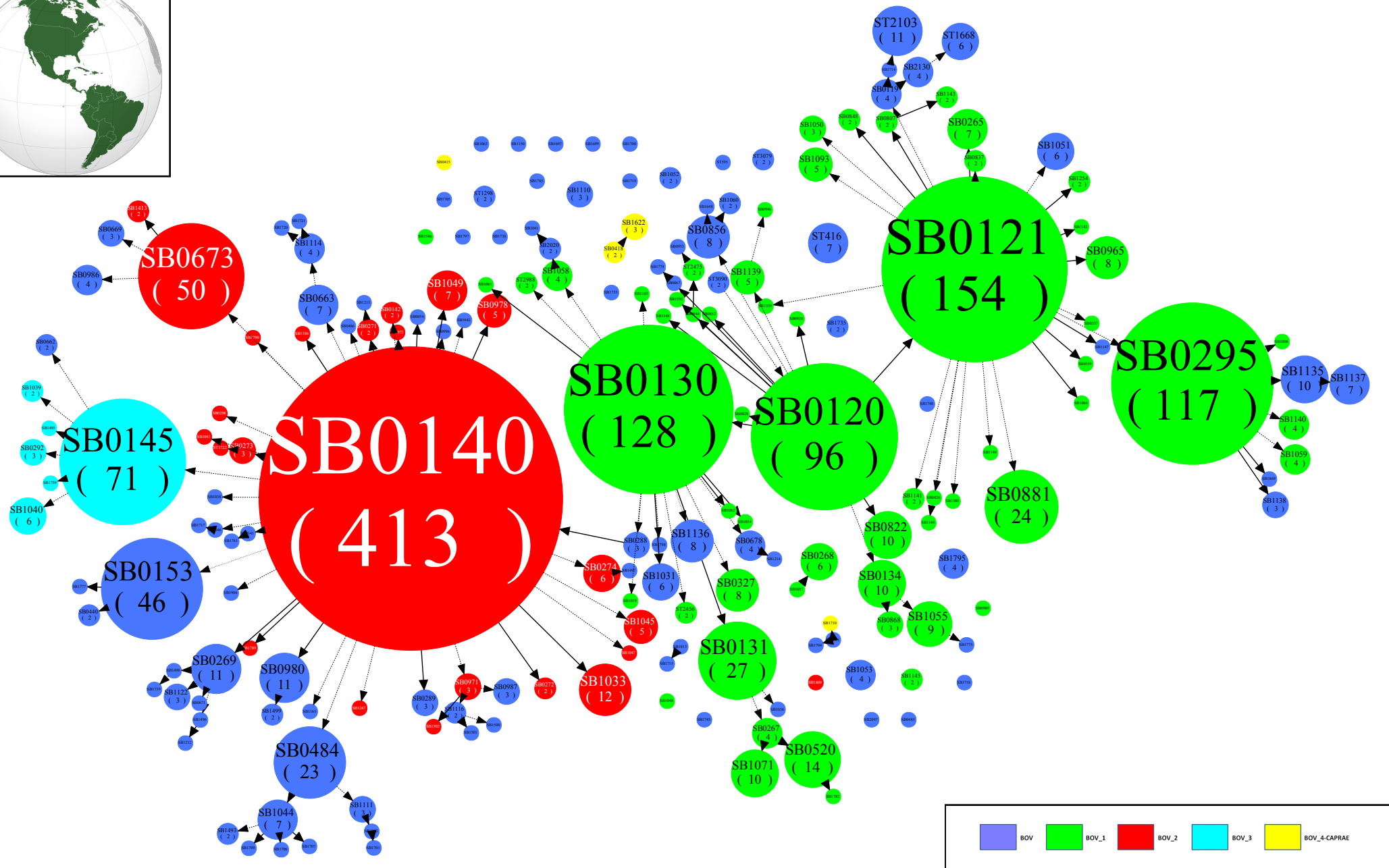

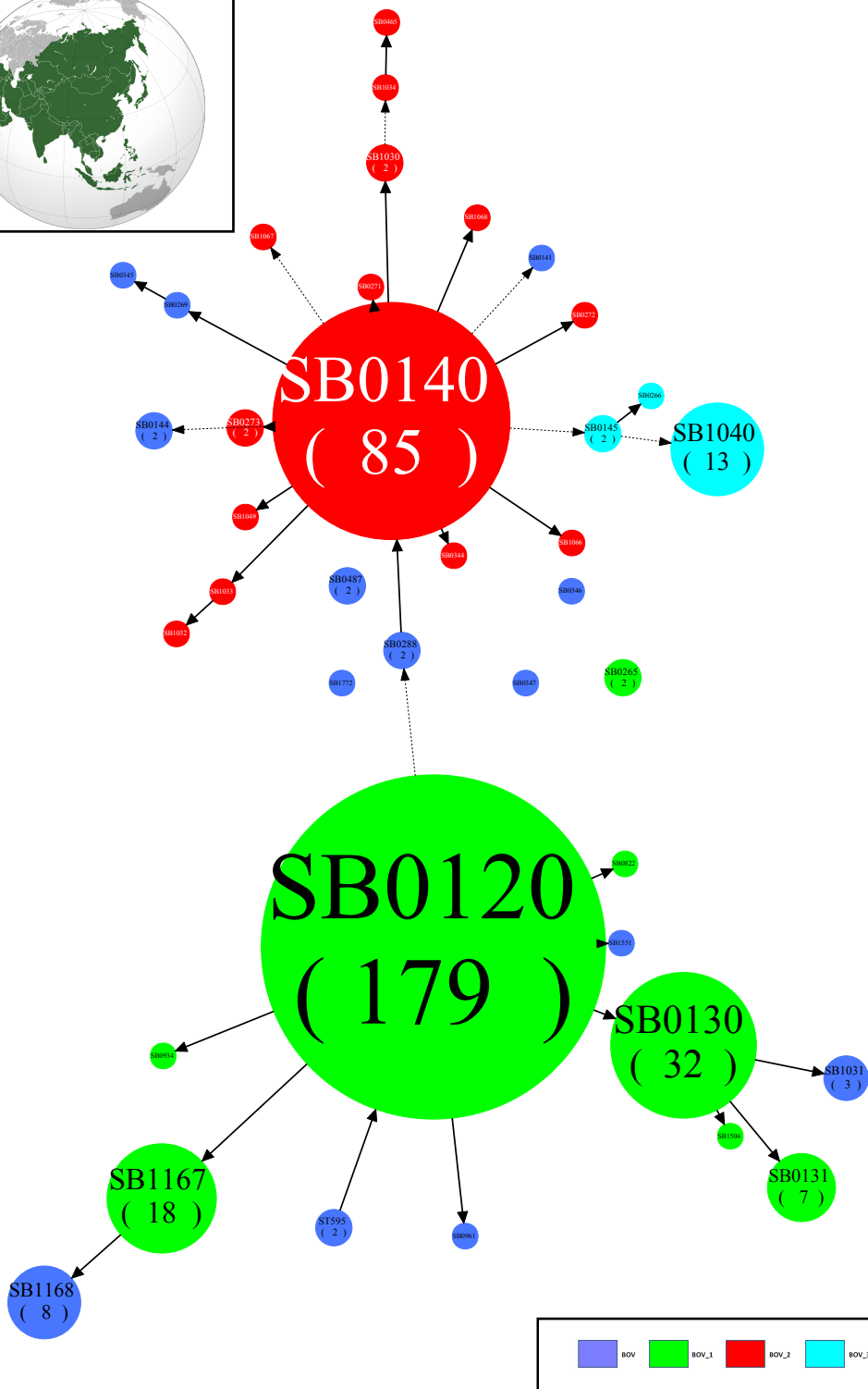

AUSTRALASIA (n=105)

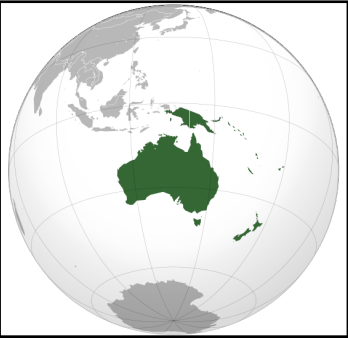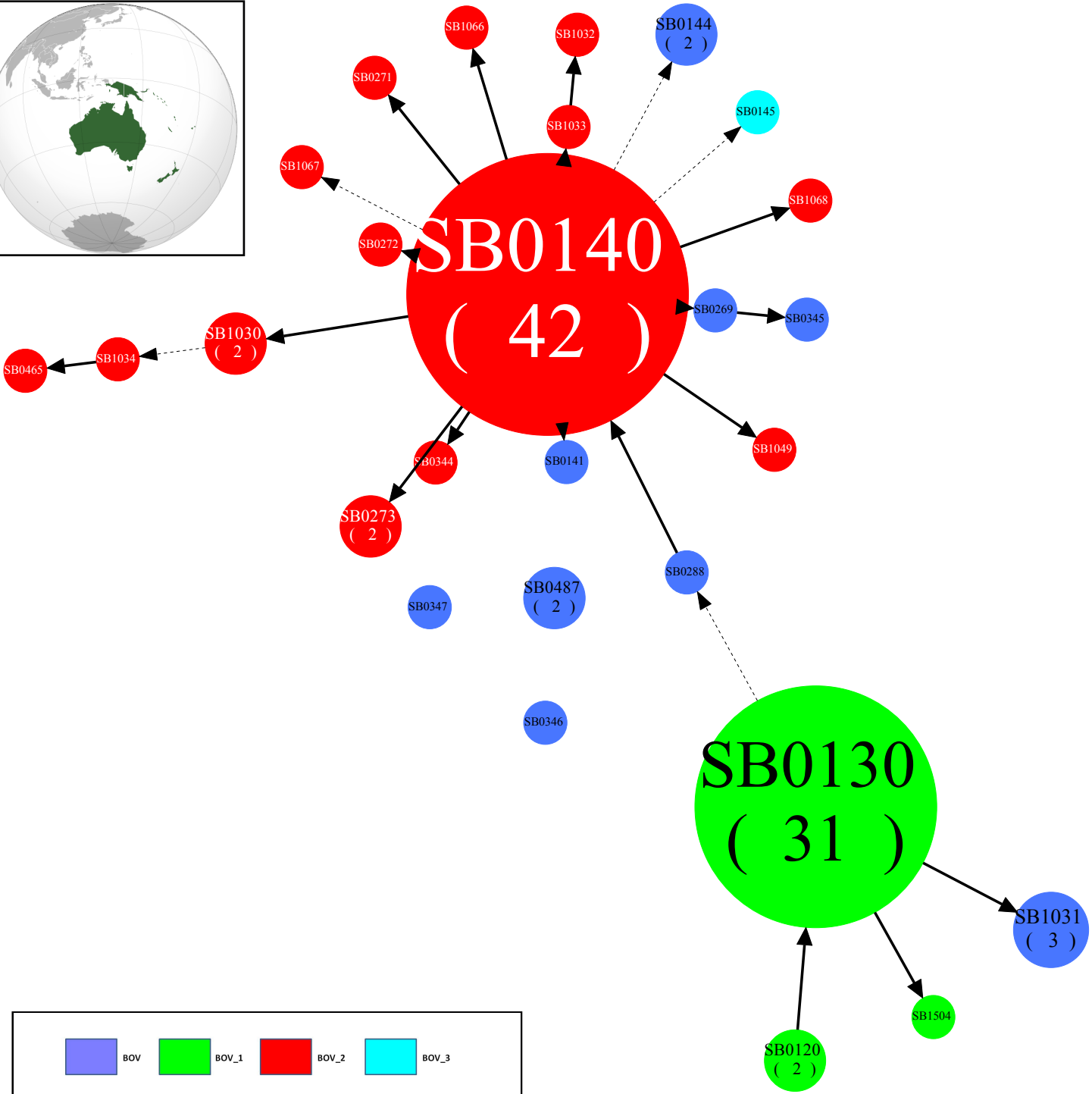

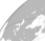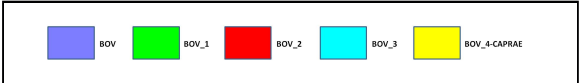

Supplement: baab081_Supp [file baab081_supp.zip › Supplementary_File_2_Spoligoforests_by_continent.pdf]
